# Layered double hydroxides for corrosion-related applications—Main developments from 20 years of research at CICECO

**DOI:** 10.3389/fchem.2022.1048313

**Published:** 2022-11-18

**Authors:** João Tedim, Tiago L. P. Galvão, Kiryl A. Yasakau, Alexandre Bastos, José R. B. Gomes, Mario G. S. Ferreira

**Affiliations:** ^1^ CICECO-Aveiro Institute of Materials, Department of Materials and Ceramic Engineering, University of Aveiro, Campus Universitário de Santiago, Aveiro, Portugal; ^2^ CICECO-Aveiro Institute of Materials, Department of Chemistry, University of Aveiro, Campus Universitário de Santiago, Aveiro, Portugal

**Keywords:** layered double hydroxides, corrosion, nanocontainer, coatings, films, corrosion detection, concrete, computational design

## Abstract

This work describes the main advances carried out in the field of corrosion protection using layered double hydroxides (LDH), both as additive/pigment-based systems in organic coatings and as conversion films/pre-treatments. In the context of the research topic “Celebrating 20 years of CICECO”, the main works reported herein are based on SECOP’s group (CICECO) main advances over the years. More specifically, this review describes structure and properties of LDH, delving into the corrosion field with description of pioneering works, use of LDH as additives to organic coatings, conversion layers, application in reinforced concrete and corrosion detection, and environmental impact of these materials. Moreover, the use of computational tools for the design of LDH materials and understanding of ion-exchange reactions is also presented. The review ends with a critical analysis of the field and future perspectives on the use of LDH for corrosion protection. From the work carried out LDH seem very tenable, versatile, and advantageous for corrosion protection applications, although several obstacles will have to be overcome before their use become commonplace.

## 1 Introduction

Corrosion can be defined as the deterioration of a metal by chemical or electrochemical reactions with the surrounding environment (Revie and Uhlig, 2008; Jones, 2014). With a wide range of use of metallic substrates in different areas of society, from infrastructures to vehicles and biomedical implants, it is not surprising that its importance can be reflected on economy, efficiency, energy, safety, and conservation factors. All these aspects touch key points in a fast-developing world, still struggling to resume global activity after the COVID-19 pandemic, in parallel with important aspects for present and future generations that are addressed in the UN Sustainability goals, including climate change and circular economy.

There are several ways of preventing and protect metallic substrates against corrosion, being one of the most used, the application of protective coatings. With the increased awareness of society for human and environmental impact aspects, it became clear that several of the most effective solutions used to protect metals against corrosion lacked sufficient environmental friendliness. Hence, corrosion inhibitors such as those derived from Cr(VI) species were prohibited and, as a result, the whole field of science and corrosion engineering has been struggling to find equally effective additives and pigments, but less toxic and harmful, to replace chromates (Zheludkevich, 2009).

In this short review we address one of the materials which have been proposed and developed during the last 2 decades as promising for corrosion protection: layered double hydroxides (LDH). In the context of the Research Topic “Celebrating 20 Years of CICECO–Aveiro Institute of Materials”, this review is mostly focused on the main works developed in SECOP–Surface Engineering and Corrosion protection group over the last 15 years, giving proper context of those works with respect to current literature whenever relevant. Table 1 summarizes the main works which SECOP group at CICECO carried out over the years, some of them in collaboration with other groups. In addition, readers are entitled to read more general and recent reviews on LDH applied to corrosion protection elsewhere (Bouali et al., 2020b; Mir et al., 2020; Tabish et al., 2021; Leal et al., 2022).

**TABLE 1:** Overview of CICECO’s functional LDH for corrosion-related applications.

LDH	Active species	Pigment/film	Type of coating/substrate	References
Zn (2)Al	nitrate	Pigment	AA2024-T3	Poznyak et al. (2009)
Zn (2)Al	quinaldate	Pigment	AA2024-T3	Poznyak et al. (2009)
Zn (2)Al	MBT^-^	pigment	AA2024-T3	Poznyak et al. (2009)
Mg (2)Al	nitrate	pigment	AA2024-T3	Poznyak et al. (2009)
Mg (2)Al	quinaldate	pigment	AA2024-T3	Poznyak et al. (2009)
Mg (2)Al	MBT^-^	pigment	AA2024-T3	Poznyak et al. (2009)
LDH intercalated with different organic corrosion inhibitors				
Zn (2)Al	vanadate	pigment	AA2024-T3	Tedim et al. (2010)
Zn (2)Al	phosphate	pigment	AA2024-T3	Tedim et al. (2010)
Zn (2)Al	MBT^-^	pigment	AA2024-T3	Tedim et al. (2010)
Enhancement of active corrosion protection by combination of inhibitor-loaded LDHs				
Zn (2)Al	vanadate	pigment	AA2024-T3	Zheludkevich et al. (2010)
Mg (2)Al	vanadate	pigment	AA2024-T3	Zheludkevich et al. (2010)
Active protection coatings based on LDH nanocontainers intercalated with corrosion inhibitors				
Zn (2)Al	nitrate	film	AA2024-T3	Tedim et al. (2011)
Zn (2)Al	pyrovanadate	film	AA2024-T3	Tedim et al. (2011)
Nanostructured LDH-container layer grown on top of AA2024 for corrosion protection				
Zn (2)Al	nitrate	pigment	polymer layer	Tedim et al. (2012)
Demonstration of LDH aggressive anion trapping ability in active protective coatings				
Zn (2)Al	MBT^-^	pigment	Galvanised steel	Montemor et al. (2012)
Combination of LDH and cerium molibdate nanocontainers filled with MBT corrosion inhibitor				
Zn (2)Al	vanadate	film	AA2024-T3	Tedim et al. (2013)
Dependence of the performance of LDH conversion films on the metal pre-treatment				
Zn (2)Al	nitrate	film	AA2024-T3	Tedim et al. (2014)
Zn (2)Al	vanadate	film	AA2024-T3	Tedim et al. (2014)
Influence of preparation conditions of LDH conversion films on their protection performance				
Zn (2)Al	MBT^−^ and Ce^3+^	pigment	AA2024-T3	Carneiro et al. (2015)
MBT was intercalated, while Ce^3+^ was fixed between polyelectrolyte layers on the LDH surface				
Zn (2)Al	nitrate	film	AA2024-T3	Tedim et al. (2016)
Zn (2)Al	vanadate	film	AA2024-T3	Tedim et al. (2016)
SVET analysis of the corrosion protection of LDH conversion films grown on AA2024				
Zn (2)Al	nitrate	pigment	-	Galvão et al. (2016a)
Density functional theory simulation of LDH-NO_3_ XRD and interlayer structural features				
Zn (2)Al	vanadate	film	AA2024-T3	Kuznetsov et al. (2016)
Sealing of tartaric sulfuric anodized AA2024 with nanostructured LDH layers				
Zn (2)Al	nitrate	film	AA2024-T3	Galvão et al. (2017)
Density functional theory simulation of LDH based conversion films				
Zn (2)Al	vanadate	film	PEO coatings/AA2024	Mohedano et al. (2017)
An LDH layer was grown on PEO coatings for AA2024 and loaded with a corrosion inhibitor				
Zn (2)Al	MBT	pigment	-	Kuznetsova et al. (2017)
Study of the antimicrobial activity of LDH-MBT				
Zn (2)Al	MBT	pigment	-	Martins et al. (2017b)
Study of toxicity induced by LDH-MBT to clams				
Zn (2)Al	Cu and Zn pyrithiones	pigment		Gutner-Hoch et al. (2018)
				Gutner-Hoch et al. (2019)
Study of toxicity induced by LDH-booster biocides to green microalgae, diatoms and mussels, brine shrimps, sea urchins				
Zn (2)Al	MBT^-^	pigment	AA2024-T3	Abdolah Zadeh et al. (2018)
Synergetic active corrosion protection with a cerium doped Y-type zeolite				
Zn (2)Al	nitrate	pigment	AA2024-T3	Yasakau et al. (2018)
Zn (2)Al	vanadate	pigment	AA2024-T3	Yasakau et al. (2018)
Mg (2)Al	chloride	pigment	-	Pérez-Sánchez et al. (2018)
Mg (2)Al	nitrate	pigment	-	Pérez-Sánchez et al. (2018)
Mg (2)Al	carbonate	pigment	-	Pérez-Sánchez et al. (2018)
Zn (2)Al	chloride	pigment	-	Pérez-Sánchez et al. (2018)
Zn (2)Al	nitrate	pigment	-	Pérez-Sánchez et al. (2018)
Zn (2)Al	carbonate	pigment	-	Pérez-Sánchez et al. (2018)
Development of a classic molecular dynamics framework to explore LDHs				
Zn (2)Al	MBT	film	AA2024-T3	Neves et al. (2019)
LDH-MBT film modified with hydrophobic silane with anticorrosion and antimicrobial properties				
Zn (2)Al	nitrate	film	zinc	Mikhailau et al. (2019)
One-step synthesis and growth mechanism of LDH based conversion coatings on zinc				
Mg (2)Al	phosphate	pigment	cast iron	Vieira et al. (2019)
Mg (2)Al	Ce^3+^/phosphate	pigment	cast iron	Vieira et al. (2019)
Cast iron corrosion protection with LDHs				
Zn (2)Al	nitrate	pigment	-	Novell-Leruth et al. (2020)
Zn (2)Al	MBT^-^	pigment	-	Novell-Leruth et al. (2020)
Molecular dynamics simulation of the structure and hydration of LDH-NO_3_ and LDH-MBT				
Zn (2)Al	nitrate	film	AA2024-T3	Bouali et al. (2020a)
Zn (2)Al	nitrate	film	zinc	Bouali et al. (2020b)
Synchrotron high-resolution XRD was used to follow the anion exchange of nitrate by chloride				
Zn (2)Al	nitrate	pigment	Steel-Reinforced Concrete	Gomes et al. (2020)
Zn (2)Al	nitrite	pigment	Steel-Reinforced Concrete	Gomes et al. (2020)
LDHs for controlling the corrosion of steel in reinforced concrete				
Zn (2)Al	nitrate	film	zinc	Iuzviuk et al. (2020)
*In situ* synchrotron XRD was used to follow the substitution with chloride, sulfate and vanadate				
Zn (2)Al	Hexacyanoferrate	pigment	carbon steel	Wilhelm et al. (2020)
Mg (2)Al	Hexacyanoferrate	pigment	carbon steel	Wilhelm et al. (2020)
Hexacyanoferrate-intercalated LDHs for steel corrosion detection				
Zn (2)Al	nitrite	pigment	cement	Mir et al. (2021)
Stability and chloride entrapping capacity of ZnAl-NO_2_ in a cement model system				
Mg (2)Al	Ce^3+^	pigment	AA2024-T3	Vieira et al. (2022)
Mg (2)Al-Ce to prolong the service life of aluminum alloys				

## 2 Layered double hydroxides

### 2.1 Structure and properties

LDH are a class of versatile materials with useful properties associated with their anion exchange abilities for a wide range of materials’ applications including adsorbents, catalysts and its support, ceramic precursors, drug carriers, corrosion inhibitor carriers, supercapacitors, nanocomposites, energy conversion and storage, carbon dioxide sequestration, among many others (Wang and Ohare, 2012; Tian et al., 2016).

LDH are lamellar compounds having molecular formula [M(II)_1−*x*_M(III)_*x*_ (OH)_2_]^*x*+^(A_*x*/*n*_^*n*−^)*.m*H_2_O, where *x* ranges from 0.22 to 0.33, M is a metal and A^*n*−^ is a *n* ^−^ valent anion. They are also called hydrotalcites since their structure is like that of the natural hydrotalcite, i.e., Mg_6_Al_2_(OH)_16_[CO_3_]·4H_2_O. This clay consists of positively charged brucite-like layers [brucite = Mg(OH)_2_] where the cations are octahedrally coordinated with OH^−^, in slightly distorted octahedra that share their edges, forming the LDH layer, and of interlayer anions balancing the positive charge due to the partial substitution of bivalent Mg with trivalent Al and allowing the layers to stack onto one another by electrostatic forces.

**FIGURE 1:**
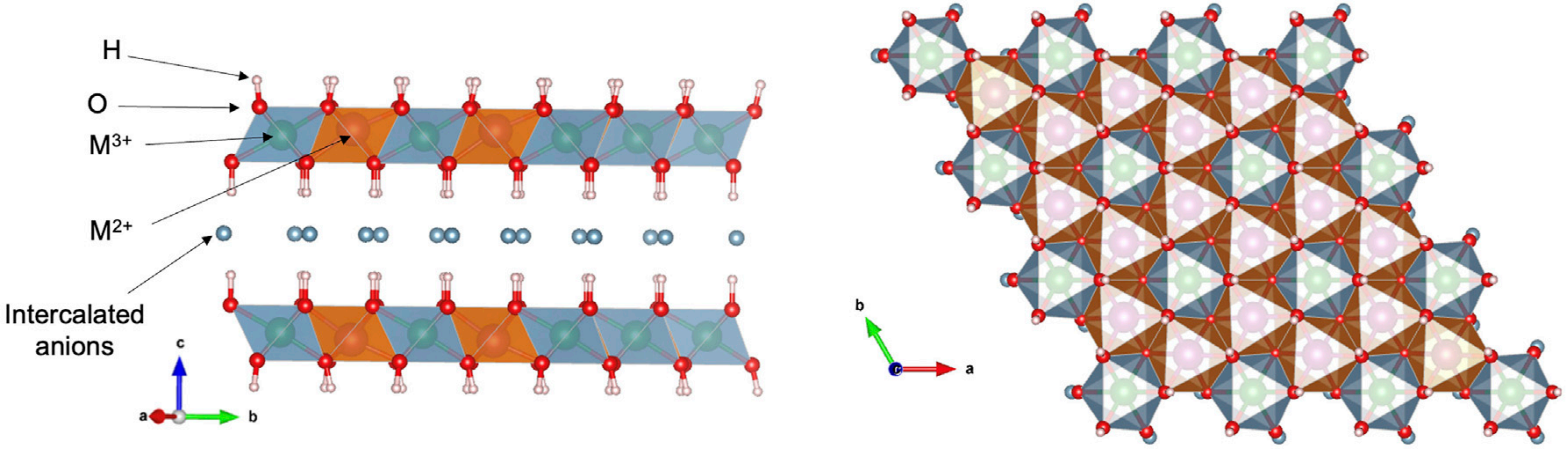
Schematic representation of the structure of a generic traditional layered double hydroxides (LDH) orthogonally and along the crystallographic axis c, depicting the distorted octahedral sheets. The figures of structural models have been constructed using VESTA software (Momma and Izumi, 2011; Leal et al., 2022). Reproduced from (Leal et al., 2022) with permission from Elsevier.

Figure 1 shows a schematic representation of a generic LDH structure having molar ratio of M^2+^:M^3+^ of 2:1, as frequently described in many minerals intercalated with hydrated carbonate anions (Guo et al., 2010; Wang and Ohare, 2012). The positions that the M^3+^ and M^2+^ octahedra adopt in the layer are well defined in the crystal lattice, although it depends on the ratio between the trivalent and the divalent species. In this case, the M^2+^ octahedra are in such configuration that resembles a dioctahedral-like sheet, in which the M^3+^ ions would occupy its vacancies (Leal et al., 2022).

The peculiar property of LDH is the possibility to exchange the interlayer anions; for this reason, they are also named anionic clays. LDH possess sandwich-like structure in which negative inorganic or organic anions are sandwiched into positively charged metal layers in a repeating manner. The hydroxide layers could be fabricated with combination of different divalent (Cd^2+^, Mn^2+^, Fe^2+^, Pb^2+^) and trivalent (Al^3+^, Cr^3+^, Fe^3+^) metal ions. The structural characteristics of the LDH allow the possibility to use a wide variety of intercalation compounds either by modification of the chemical composition of the hydroxide layer or by chemical/structural modification of the interlayers. The particle size could change from nanometer (nm) to micrometer (μm), depending on synthesis conditions.

Depending on the application, several methods (Bouali et al., 2020b) were reported for the formation of LDH powders relying mostly on chemical reactions (Levin et al., 1996; Takei et al., 2014), namely, co-precipitation, sol-gel method using alkoxides and/or acetylacetonate as starting precursors (Prinetto et al., 2000), urea hydrolysis method (Hibino and Ohya, 2009), hydrothermal method (Zhi and Guo, 2005), reformation (Theiss et al., 2012) and mechanical milling (Qu et al., 2019). The co-precipitation method is the most straightforward and commonly applied. This method could involve one-step synthesis only, or be followed by anion exchange reactions between the interlayer anion and the targeted anion (two-step synthesis).

Typically, the synthesis by co-precipitation can be achieved at either variable pH (titration co-precipitation) or constant pH conditions. The latter option is preferable to obtain pure, crystalline LDH. During the reaction, the pH of the solution is kept constant by the simultaneous addition of an alkaline solution (e.g., NaOH or NH_3_•H_2_O) together with the precursor solution of mixed metal salts (metals that will be part of the LDH). Usually, an alkaline solution is chosen according to the corresponding metal salts and the desired anion to be intercalated between the LDH galleries. Additionally, since it is difficult to avoid the presence of CO_2_ in air, it is further advised to work under nitrogen or argon flow to avoid the formation of LDH structures with intercalated carbonate species, if these are not desirable.

The LDH product of the synthesis by co-precipitation relies upon a crucial control of the pH of the reaction medium, the concentration and nature of both alkaline and metal precursor solutions (besides the molar ratio of the metal cation itself), the temperature and aging time (Bouali et al., 2020b).

In the attempts to confer corrosion protection to metallic substrates “smart” active corrosion protective systems have been searched to replace chromate and pre-treatment containing coatings, due to the known health problems that Cr(VI) can originate. There are several reviews available in the literature listing numerous materials developed to replace chromates, including polymeric microcapsules, oxide nanoparticles (Zheludkevich, 2009; Zheludkevich et al., 2012; Zhang et al., 2018) and graphene (Kulyk et al., 2022), just to mention a few. Among those that have received the most attention are LDH. The reason behind the choice of selecting LDH for corrosion protection, to the detriment of other nanocontainers (Bouali et al., 2020b) can be explained by the fact that LDH have the remarkable option to be used both in form of anticorrosion pigments incorporated into a coating system (Zheludkevich et al., 2012), as well as a conversion coating on the metal (Tedim et al., 2016). This means that it can be easily adapted according to different requirements. On one hand, LDH can be used as a powder/slurry to confer additional active protection properties to a barrier coating by the addition of a self-healing functionality (Zheludkevich et al., 2010). On the other hand, LDH can be applied directly to the metal surface as a conversion film/coating, similarly to Cr(VI) technology, if there is a need for the first coating layer (close to the metal) to be an active corrosion protective system (Tedim et al., 2011). LDH are among the most investigated and with more application potential anion exchange nanocontainers for “smart” active corrosion protection (Zheludkevich et al., 2010; Wang and Ohare, 2012; Zhang et al., 2015).

Keeping in mind that most well-known and studied LDH are based on compositions such as ZnAl or MgAl, attempts to develop LDH-based conversion films have occurred during the last 25 years (Leggat et al., 2002; Kuznetsov et al., 2016; Visser et al., 2016; Mikhailau et al., 2019; Iuzviuk et al., 2020). The simplest *in-situ* LDH growth can be achieved by a co-precipitation process, which is an extension of the LDH powder synthesis by co-precipitation. In this case, the substrate to be treated is also one of the precursors. In the case of Al substrate, M/Al LDH films can be fabricated by the immersion of the substrate in a bath containing a metal cation M^2+^/M^+^ (Zn^2+^, Mg^2+^, Li^+^, etc.) precursor in certain conditions (pH, temperature, concentration etc.), while the Al^3+^ ions are generated by the dissolution of the Al substrate. Specific anions could be carried out through anion exchange reaction by simple immersion of the LDH treated Al alloy into a solution containing the respective species at a specific pH, concentration, and temperature.

### 2.2 Application of LDH in corrosion

**FIGURE 2:**
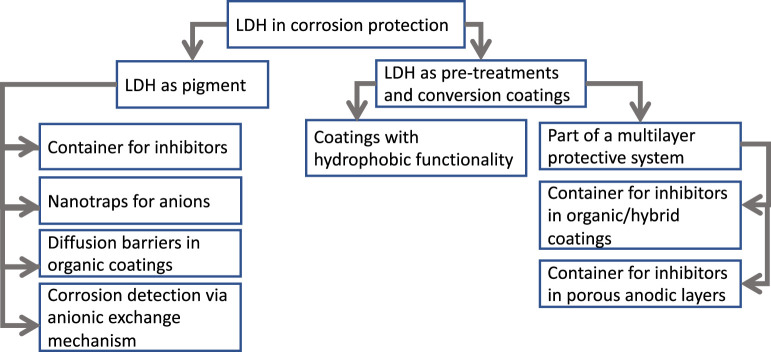
Application of LDH in corrosion protection.

The corrosion protection performance of LDH mainly comes from its structural and chemical properties, including the formation of physical protective films, presence of inhibitors contained in the LDH nanostructure and self-healing effect. The controlled release of inhibitors from LDH occurs by anion exchange, normally with chloride ions present in the aggressive medium, leading to their capture, and then a double protection effect. Figure 2 summarizes the main roles that LDH can play in the context of corrosion protection.

The breadth of works reporting layered double hydroxides-based materials, both as particle-like (so-called nanocontainers) and as conversion coatings, dates back to the mid 1990s-beginning of 2000s, with pioneering works by Buchheit (Buchheit et al., 2002; Zhang and Buchheit, 2002; Buchheit et al., 2003), Williams and McMurray (McMurray and Williams, 2004; Williams and McMurray, 2004). Both conversion coatings and active corrosion protection pigments based on LDH aimed at replacing hexavalent Cr, particularly in applications where Cr(VI)-based pre-treatments and primers had been the main solution for anti-corrosion protection: the aerospace industry (Twite and Bierwagen, 1998). Hence, it is not surprising that these early works have focused mostly on aluminum alloys.

With respect to the use of LDH as anticorrosion pigments in organic coatings, Williams and McMurray investigated nitrate-, carbonate- and chromate-containing Mg-Al LDH, which were added to a polyvinyl butyral (PVB) coating applied onto AA2024-T3 substrates to investigate how these pigments could affect the development of filiform corrosion (McMurray and Williams, 2004). In all the LDH-modified coatings, the rates of coating delamination were reduced and the inhibiting efficiency of the LDHs was found to be dependent on the nature of the intercalating anion, with the chromate-containing LDH displaying the best result. In another work by the same authors (Williams and McMurray, 2004), different organic corrosion inhibitors, namely benzotriazole, ethyl xanthate and oxalate were anion-exchanged in LDH and their effect on filiform corrosion of a PVB-coated AA2024-T3 substrate were surveyed. Benzotriazole was found to render the best inhibition efficiency.

In an independent contemporary study by Buchheit and colleagues (Buchheit et al., 2003), the preparation of Zn-Al LDH intercalated with decavanadate was described and the effect of this LDH material was surveyed in an epoxy resin coating. The resulting Zn-Al LDH was prepared firstly by modifying the vanadate speciation from meta to decavanadate form and then obtained the LDH material by direct coprecipitation in a carbonate-free environment. The structural analysis performed by XRD, unambiguously showed the expansion of galleries when decavanadate was present in the interlayer spacing between the mixed-metal hydroxide sheets, with respect to chloride-containing LDH. Release studies were also performed in 0.5 M NaCl solution, revealing leaching of both Zn and V species, with the latter ascribed to anion-exchange reaction between chlorides and vanadates. On the other hand, Zn release was claimed to be associated with co-intercalation of Zn^2+^ or with formation of a hetero-polyoxometalate of Zn and V, which became subsequently immobilized in the LDH galleries. Furthermore, corrosion inhibition studies demonstrated a clear protective effect of the coatings loaded with V-containing LDH (salt spray tests and electrochemical impedance spectroscopy, EIS, studies). In addition, the same work described the possibility of using LDH as a corrosion sensing pigment as XRD analysis of LDH-vanadate (LDH-VO*_x_*) exposed to NaCl solution, directly as powder or embedded within the epoxy resin, reveals the occurrence of a second LDH phase associated with intercalation of chlorides, constituting a way of detecting the earlier uptake of electrolyte by the coating (corrosion sensing).

As mentioned earlier, the works by Buchheit’s group have not been restricted to the use of LDH as pigments in organic coatings. They also laid the foundations for some of the most trending works in terms of active protection, Cr-free, surface pre-treatments and Li-based pigments for organic coatings (Liu et al., 2016). In their works, Buchheit and colleagues used different oxidizing bath chemistries to obtain LiAl LDH (Buchheit et al., 1994; Buchheit et al., 2002; Zhang and Buchheit, 2002), in an attempt to reduce processing times for coating formation and increase in corrosion resistance. The results showed that nitrate/persulfate bath chemistries gave rise to better corrosion resistance when compared to carbonate or nitrate-only based chemistries. The healing characteristics of the obtained coatings was ascribed to competition between chloride attack and sealing of coatings formed under oxidizing chemistries. In another work (Buchheit et al., 2002) a Ce LDH conversion coating was investigated as a potential self-healing protective system using a simulated scratch cell. The healing effect of this system was associated to the introduction of Ce in the LDH as a soluble, high-oxidation state species which in presence of solution dissolve and Ce^4+^ is reduced and precipitated as Ce^3+^ compound on the exposed Al sites.

#### 2.2.1 LDH as reservoirs for corrosion inhibitors in coatings

##### 2.2.1.1 New LDH compositions for active corrosion protection

The first few works describing layered double hydroxides in the literature revealed promising properties of this class of materials for corrosion protection. They were reported right after the concept of self-healing being described by White et al. (White et al., 2001) and, as a result, the concept was also adapted to the field of anti-corrosion protective coatings. Healing in this context does not necessarily imply a healing of defects in polymeric systems *via* recovery of structural integrity of the protective coating only (e.g., healing by polymerization), but also the controlled release of active species such as water-displacing hydrophobic agents and corrosion inhibitors, from the so-called *smart micro and nanocontainers* which are embedded within polymeric matrices and are able to protect the exposed surface–*functional self-healing*. Several works have reported a wide range of materials as potential systems for active corrosion protection, from mesoporous particles to polymeric microcapsules (Zheludkevich et al., 2012; Zhang et al., 2018). In this section the most relevant work published by our group using LDH as smart nanocontainers for corrosion protection is revisited.

**FIGURE 3:**
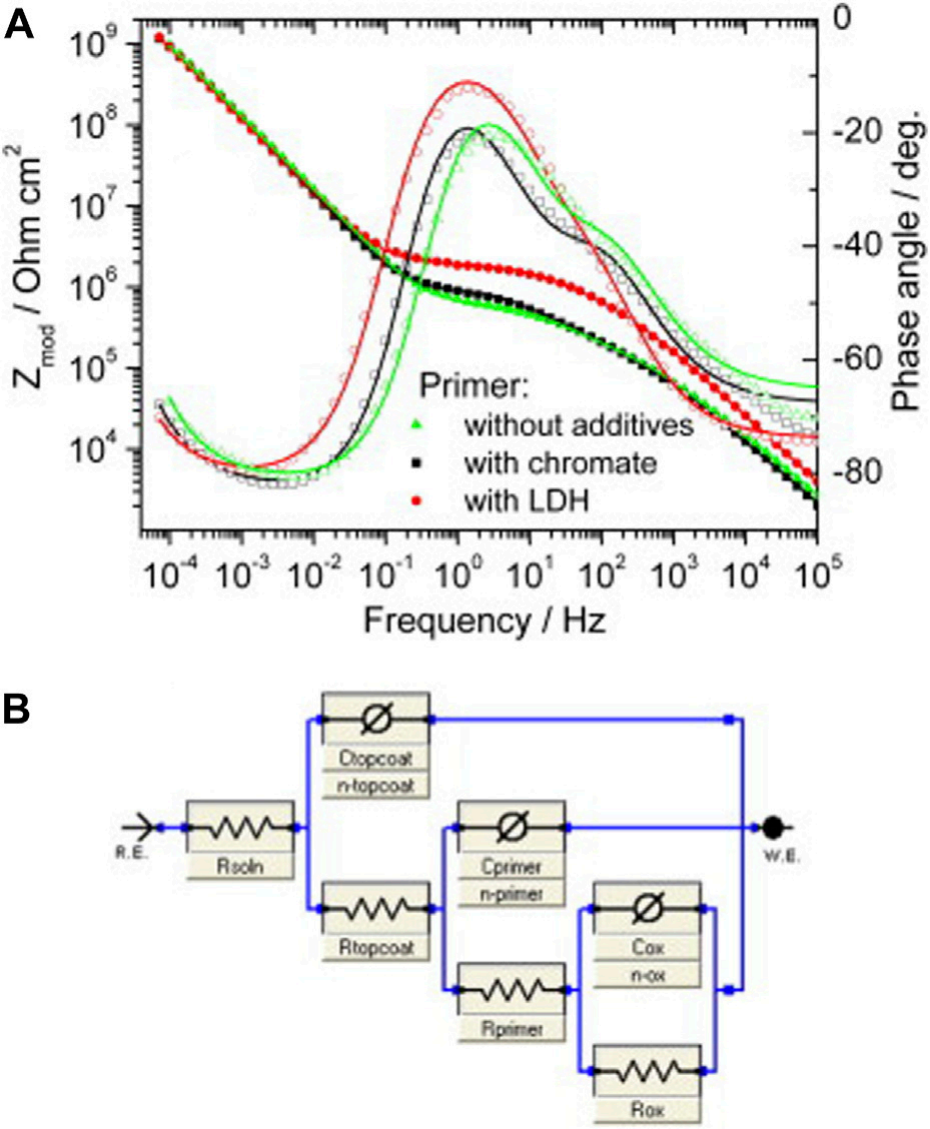
Bode plots **(A)** of AA2024 samples, coated with primer and top coat without additives, with chromate and with LDH-VO_x_, taken after 4 months of immersion in 0.5 M NaCl. Solid lines show the fitting results according to model **(B)**. Reproduced from (Zheludkevich et al., 2010) with permission from Elsevier.

The early works reported by our group on LDH have been based on ZnAl and MgAl compositions, intercalated with different corrosion inhibitors. Vanadate-intercalated ZnAl and MgAl LDH were prepared by direct co-precipitation and ion-exchange (Zheludkevich et al., 2010). Vanadates were intercalated in the pyrovanadate (V_2_O_7_^4−^) form by careful control of pH conditions. Structural studies revealed that LDH-VO_x_ prepared by ion-exchange of LDH-NO_3_ were more crystalline when compared to LDH-VO_x_ prepared by direct coprecipitation. Furthermore, ion exchange studies revealed the release of V-containing species and EIS measurements demonstrated the inhibiting properties of these LDH against corrosion of AA2024-T3 in NaCl solution. The performance of ZnAl LDH-VO_x_ prepared by ion-exchange was compared against strontium chromate pigment, using both type of pigments in an epoxy-resin primer, as part of a coating multilayer system composed of anodizing layer, water-based primer and water-based epoxy topcoat layers. EIS measurements showed that ZnAl LDH-VO_x_ lead to an increase on both the oxide resistance and pore resistance of the organic coating system, when compared to the chromate-base pigment (Figure 3). Moreover, accelerated tests were also performed. The results showed that the chromate-based paint system was better than the LDH-based paint system in terms of neutral salt spray and resistance to osmotic blistering but worst against filiform corrosion.

In a detailed XRD work by Salak and colleagues (Salak et al., 2010), the authors went deeper on understanding structural changes occurring in LDH when nitrates were exchanged with pyrovanadates. The results from this study revealed that when anion exchange occurs between nitrates and vanadates, there is a decrease on the average crystallite size of LDH, which was ascribed to mechanical fragmentation of the crystallites because of the fast anion exchange reaction.

In another work, different organic inhibitors, namely quinaldate (QA) and 2-mercaptobenzothiazolate (MBT) anions were intercalated in MgAl and ZnAl LDH by ion exchange. XRD patterns and FTIR spectra revealed the presence of these anions within the LDH galleries. Release studies performed in aqueous NaCl solutions with different concentrations demonstrated that higher amounts of MBT were released in more concentrated NaCl solutions, which is consistent with an equilibrium-driven ion-exchange mechanism (Poznyak et al., 2009). The EIS studies showed that in the beginning of immersion tests, uncoated AA2024-T3 samples exhibited relatively low impedance values, probably due to the increase of solution pH associated with the release of QA and MBT. However, as the immersion time progressed a thick layer composed of corrosion products and inhibitor protected the substrate in the presence of ZnAl LDH-MBT, leading to an overall increase of impedance in the presence of this material. These results were discussed in terms of differences between inhibiting effect rendered by QA and MBT under different pH conditions. The way MBT and benzotriazole (BTA) arrange within Zn-Al and Mg-Al LDH galleries was also evaluated in a different work by *in-situ* XRD measurements (Serdechnova et al., 2016). Herein, the authors found out that upon the formation of LDH-MBT and LDH-BTA, there is occurrence of an additional LDH-OH phase, while in the (ZnAl or MgAl) LDH-MBT and MgAl LDH-BTA phases these organic anions form a double layer arrangement with tilted orientation with respect to the metal hydroxide sheets. In the case of ZnAl LDH, exchange between nitrates and BTA was not possible due to the formation of a compound based on zinc oxide and BTA.

##### 2.2.1.2 The chloride entrapment effect

**FIGURE 4:**
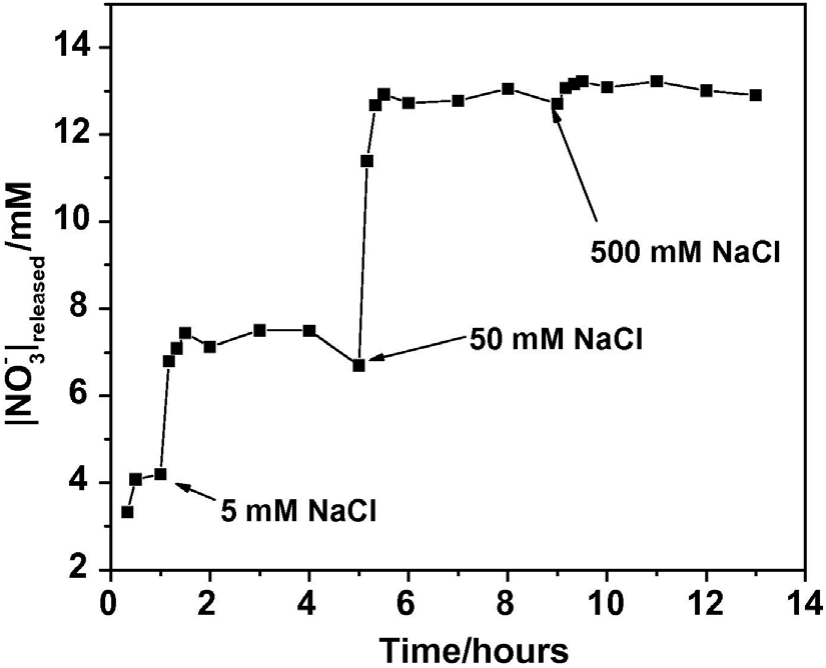
Release profiles of NO_3_− from Zn–Al–NO_3_ LDH (0.5 wt.%) in solution, in the presence of increasing amounts of NaCl. Reproduced from (Tedim et al., 2012) with permission from Elsevier.

Having been reported the in literature the effect of ion-exchange on inhibitor modified LDH, the sole effect associated with chloride entrapment within LDH and its contribution for the electrolyte permeability through a coating layer, had not been addressed before. In the work carried out by Tedim and colleagues (Tedim et al., 2012), Zn-Al LDH loaded with nitrates and chlorides were used as “empty” and “full” forms of LDH. Organic coatings loaded with ZnAl LDH-NO_3_ exhibited lower permeability to chlorides, which was demonstrated to be associated with exchange of chlorides with nitrates, while the coating with ZnAl LDH-Cl revealed a permeability to chlorides even higher than the reference coating (i.e., without LDH). Authors claimed that this increase in the coating permeability could be due to the combining effects of LDH not being able to entrap chlorides and to the disruption of coating barrier properties due to agglomeration of LDH particles during coating preparation. Equally relevant was the release/ion-exchange experiments performed, which unambiguously revealed the controlled release capacity of LDH to exchange anions with the surrounding environment, as a function of the concentration of chlorides available in the medium (Figure 4).

##### 2.2.1.3 Combination of inhibitors in micro- and nanocontainers

In a work published in 2010, the effect of combining LDH with different corrosion inhibitors, namely MBT, vanadates and phosphates, was reported (Tedim et al., 2010). Herein, authors found that when LDH intercalated with different corrosion inhibitors were combined in solution, the overall impedance associated with AA2024-T3 in 0.05 M NaCl was larger than for each individual LDH. This was the first report on the combination of different nanocontainers rendering a positive effect in terms of corrosion protection. However, when the same strategy was attempted within coatings, the results were found to be different: when both LDH-MBT and LDH-VO*_x_* were added to the primer or to the sol-gel layer, the impedance was lower than when LDH-MBT was added to the sol-gel pre-treatment and LDH-VO_x_ was added to the primer. Overall, this work revealed that compatibility between the nanocontainer and the coating matrix is of paramount importance when designing a protective coating system and that the availability of inhibitors in the pre-treatment sol-gel layer for short timescale action combined with vanadates in the primer layer to render long-term protection was a promising way of combining these two LDH.

In another work carried out in collaboration with Montemor, Kordas and colleagues (Montemor et al., 2012), LDH and cerium molybdate nanocontainers, both loaded with MBT, were combined to render corrosion protection to galvanized steel. In this work, although the inhibitor was the same, the release-driven mechanisms and release timescales were expectedly different. Several electrochemical techniques, namely the scanning vibrating electrode technique (SVET) and EIS were used to investigate the self-healing ability of epoxy-based organic coatings modified with these two types of nanocontainers. The results obtained revealed a positive effect in combining the two nanocontainers, with LDH-MBT displaying a fast, short-term response whereas CeMo-MBT provided a long-term inhibition effect to protect metallic substrate. More recently, a joint work with Garcia and colleagues demonstrated that the combination of LDH-MBT with a Ce(III)-loaded zeolite provided superior protection to AA2024-T3, when the micro and nanocontainers were both added to the same water-based epoxy coating in a specific proportion (10:90) between LDH and the zeolite (Abdolah Zadeh et al., 2018).

**FIGURE 5:**
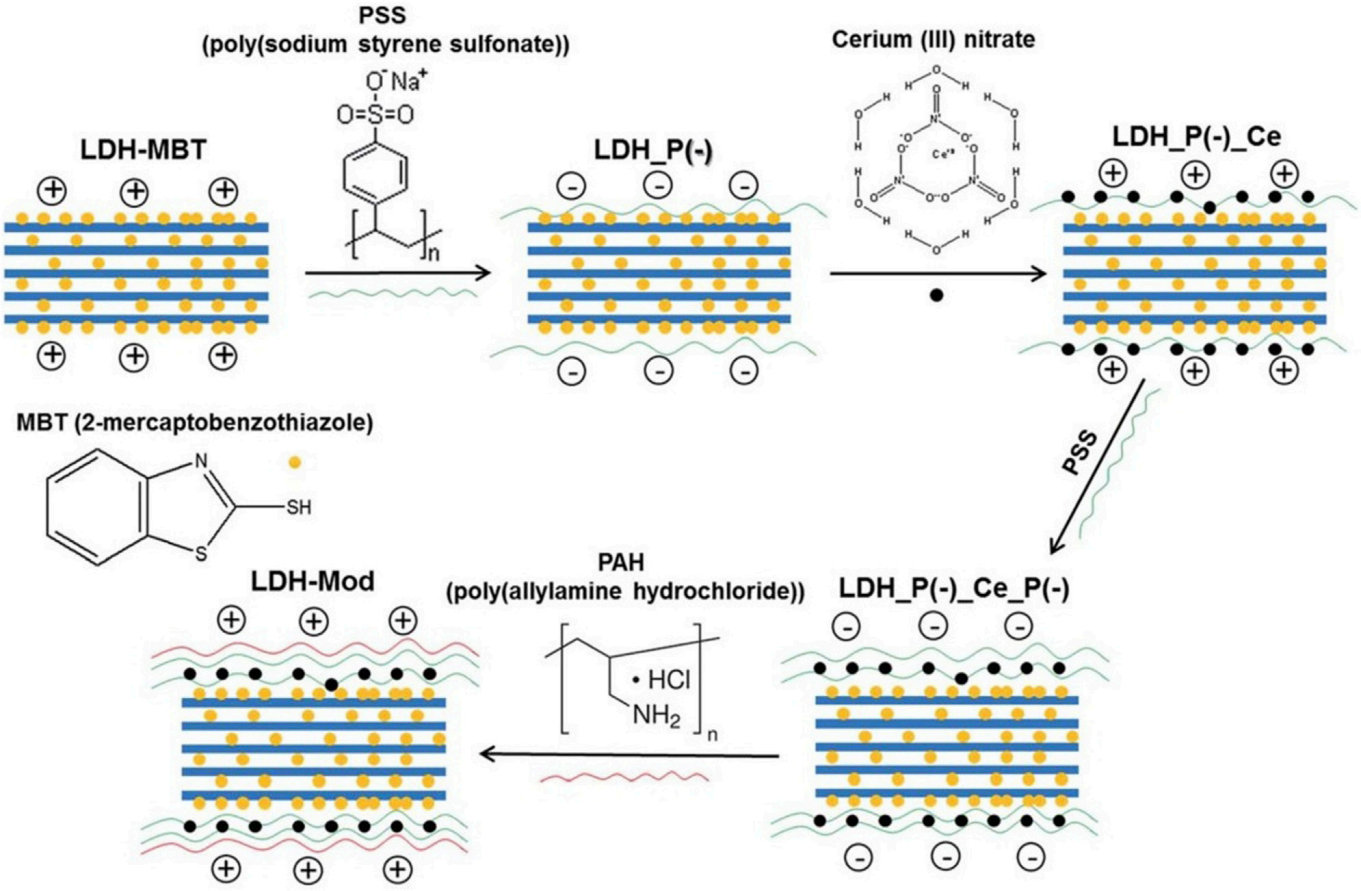
Approach used in the surface modification of LDH. Reproduced from (Carneiro et al., 2015) with permission from the Royal Society of Chemistry.

Another strategy that was explored using LDH and combination of different corrosion inhibitors relied on the surface modification of LDH with polyelectrolyte shells (Carneiro et al., 2015). It is well-known in the literature that polyelectrolytes are sensitive to changes in pH, which can be used as a triggering condition for release of corrosion inhibitors, as anodic and cathodic reactions associated with corrosion processes may lead to local pH changes (Shchukin et al., 2006). Hence, we decided to use LDH-MBT modified with polyelectrolyte shells, between which Ce^3+^ was immobilized (Figure 5). The EIS results revealed that the modification of LDH-MBT with polyelectrolyte shells had to main effects: first, there was an increase in the impedance magnitude of AA2024-T3 directly exposed to the these modified LDH, when compared to LDH-MBT, which was interpreted as a combination of having the two inhibiting species being released from the nanocontainer; second, the effect of polyelectrolytes on not letting the pH in solution increase as much as when MBT was released from LDH-MBT, as shown previously (Poznyak et al., 2009), thus contributing for an high stability of the native aluminum oxide layer. Furthermore, the presence of the polyelectrolyte shells also led to a change in the release mechanism of MBT, with MBT being released more extensively under alkaline conditions rather than more concentrated NaCl solutions. Finally, the polyelectrolyte shells contributed to a better compatibility of the LDH with a sol-gel coating system used as model formulation in this work, by reducing the exposure of the sol-gel matrix to MBT and Ce^3+^ during coating preparation.

##### 2.2.1.4 Sonication route for the synthesis of LDH

One of the most used routes for the synthesis of LDH is based on direct coprecipitation of a mixed metal salts solution under controlled pH, followed by hydrothermal treatment for several hours to promote crystallization of the LDH. However, for the sake of industrial production, any process that can lead to shorter production times and reduction of water consumption is welcomed. Recently, Salak and colleagues developed a sonication-based procedure to produce MgAl LDH. In a joint work with the group of Kareiva and co-workers (Sokol et al., 2019), they obtained MgAl LDH-H_2_PO_4_ *via* a sol-gel route, combined with successive anion-exchange processes between OH^−^ and Cl^−^ and between Cl^−^ and H_2_PO_4_^−^. They observed that the use of ultrasound treatment with 1.5 kW power accelerated the anion-exchange step. A similar approach was applied to obtain a MgAl, Ce LDH-PO_4_. The resulting LDH material was studied as an anti-corrosion nanomaterial for corrosion inhibition of cast iron (Vieira et al., 2019). In another study, the authors applied the sonication treatment during the crystallization step for 5 min to obtain a MgAl, Ce LDH-NO_3_ by co-precipitation. When exposed to UV radiation, the obtained material was found to degrade and the release of Ce^3+^ was accelerated (Vieira et al., 2022).

#### 2.2.2 LDH: From nanocontainers to pre-treatments

LDH nanocontainers have proven to be an effective means of enhancing the anticorrosion capabilities of organic coatings applied to metallic substrates. Despite that, there are several drawbacks associated with the use of nanocontainers in coatings. For instance, agglomerates formed when mixing nanocontainers with the liquid coating formulation may disrupt the barrier properties of the coating matrix in which the particles are distributed. If the coating matrix has high barrier properties LDH could effectively lower the barrier properties of such coatings leading to faster deterioration of the protection system. When the nanocontainers are located far away from the metallic surface the transport of the inhibitive species from the LDH to the metallic surface may be limited due to diffusion. However, if LDH are distributed close to the metallic surface, it will be much easier for the inhibiting species to reach the surface and provide inhibiting action. One of the ways to achieve this objective is by surface modification of a metallic surface *via* conversion films based on LDH.

In general, there are more methodologies to produce LDH coatings than LDH powders, and some of them are often separated into different groups (Guo et al., 2018; Tabish et al., 2021; Cao et al., 2022): *in situ* hydrothermal treatment, steam coating (Ishizaki et al., 2013; Kamiyama et al., 2016), spin coating (Zhang et al., 2008), co-precipitation (Zhou et al., 2015; Zhou et al., 2017), electrochemical deposition (Indira and Vishnu Kamath, 1994; He et al., 2020). The *in situ* hydrothermal methods are commonly used for the synthesis of LDH films on a broad range of metallic substrates using different water chemistry, concentration, pH of solutions, and temperature conditions. Typically, the solutions for LDH growth contain metal salts precursors and auxiliary salts and the growth is done at a high temperature directly at the metal surface (Buchheit et al., 2002). For the hydrothermal method, specific conditions are used i.e., temperature >100°C and pressure >1 bar under which LDH growth is performed most notably on Al alloys (Mohammadi et al., 2021) and Mg alloys (Wang et al., 2010; Ye et al., 2018). When there is slow kinetics of dissolution of metallic substrates that supply the cations for the LDH growth, organic and inorganic complexants e.g., NH_3_ (Guo et al., 2009; Lei et al., 2013), nitrilotriacetic acid (NTA) or ethylenediaminetetraacetic acid (EDTA) (Shulha et al., 2018) can be used to control the concentration of soluble metal species in the solution. The steam growth method (Ishizaki et al., 2013; Kamiyama et al., 2016) was proposed as an alternative to the classical hydrothermal method. Its main difference compared with the latter is that the metallic samples are placed in a hydrothermal capsule above the liquid level, so the steam reacts with the substrate surface forming the LDH. Next, the methods such as spin coating and co-precipitation often employ the hydrothermal treatment as part of the process, however, the obtained coatings lack necessary adhesion towards the metal surface, which prevents their application in corrosion protection. Although the electrochemical deposition of LDH coatings has been known for decades (Indira and Vishnu Kamath, 1994), there are shortcomings due to fast hydrogen evolution kinetics. Nonetheless, there are reports on the efficiency of corrosion protection of these LDH coatings (Wu et al., 2014; He et al., 2020). In more complex cases the various methods can be combined, which was exploited by some researchers to develop multistep LDH coating formation routes (Chen et al., 2012; Wu et al., 2017).

Buchheit et al. presented one of the first attempts to grow *in situ* hydrotalcite (HT) films on metallic surfaces such as galvanized steel and AA 2024 (Buchheit et al., 1994; Buchheit et al., 2002; Zhang and Buchheit, 2002; Buchheit and Cuan, 2004). It is noteworthy that HT is essentially a LDH and it has a general formula Mg_6_Al_2_CO_3_(OH)_16_·4H_2_O. The XRD patterns collected on samples coated with various HTs do show typical reflections (003) and (006) belonging to the layered structure of LDH (Buchheit and Cuan, 2004). Albeit HT coatings demonstrate improved corrosion protective properties HT has poor anionic exchange capabilities and cannot contribute to building active corrosion protective coatings based on intercalation of corrosion inhibitive species. Therefore, more elaborate synthesis methods of LDH films were developed. Although later Hoshino et al. (Hoshino et al., 2018) developed a process in which carbonate anions incorporated in LDHs grown on galvanized steel surfaces are substituted by nitrate anions, the process involved methanol as a solvent. A more environmentally safe route is necessary.

**FIGURE 6:**
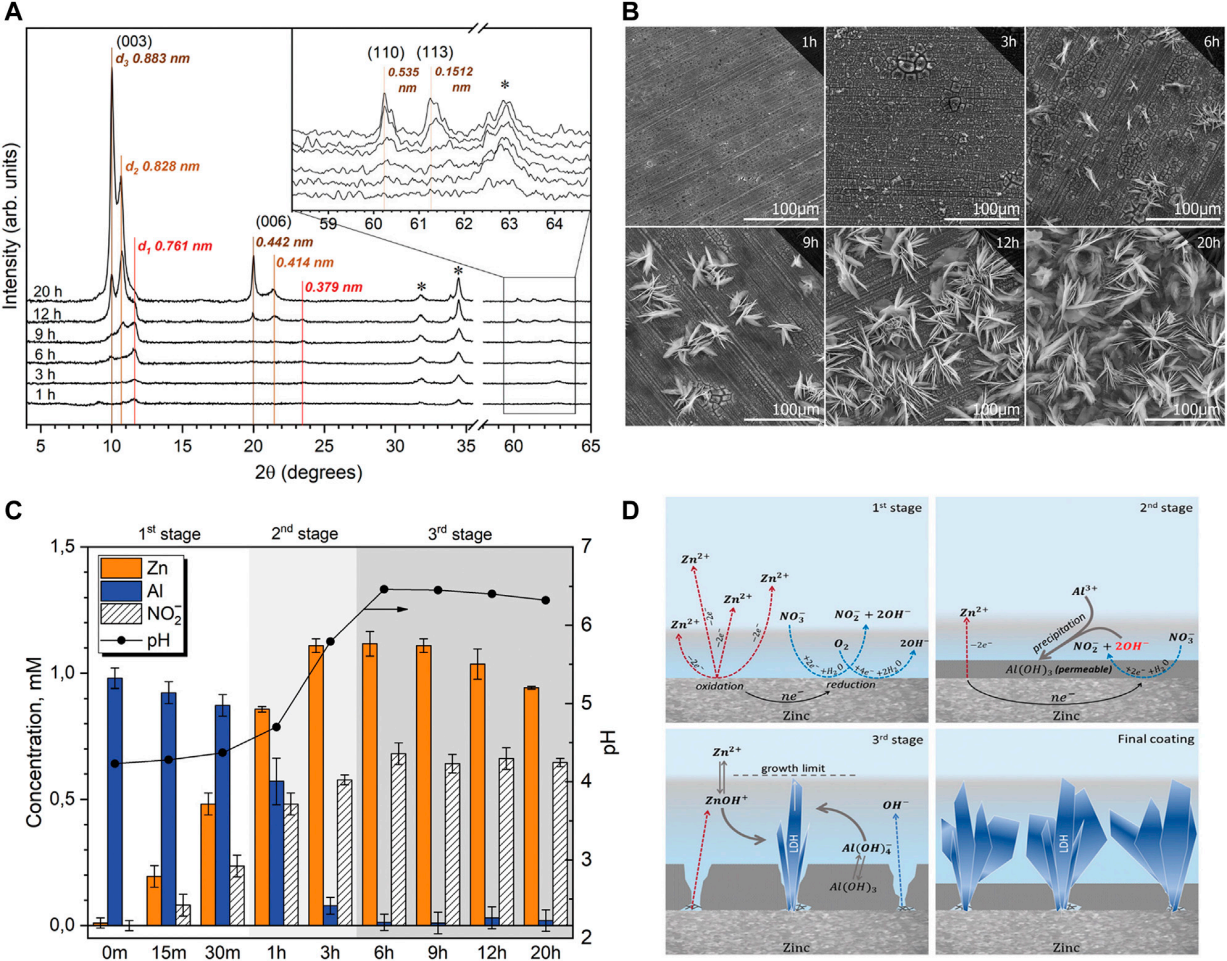
XRD patterns **(A)** and SEM micrographs **(B)** taken from zinc surface after different synthesis times; kinetics of evolution **(C)** of Al^3+^, Zn^2+^ and NO_2_^−^ concentrations in the solution, and scheme **(D)** of the LDH growth mechanism. Reproduced from (Mikhailau et al., 2019) with permission from the Royal Society of Chemistry.

One-step *in situ* synthesis of nitrate substituted LDH on zinc surface was demonstrated for the first time in a recent study (Mikhailau et al., 2019). The synthesis was carried out at 90 °C in the solution of 1 mM Al(NO_3_)_3_ and 0.1 M NaNO_3_. Figure 6A depicts the evolution of diffraction patterns taken from the zinc surface after different immersion times. Figure 6A reveals a pronounced growth of the diffraction peaks at about 10° 2*θ* and 20° 2*θ* that were assigned to different LDH phases with distinct values of the interlayer distances (basal spacing, *d*), namely *d*_1_ = 0.761 nm, *d*_2_ = 0.828 nm, and *d*_3_ = 0.883 nm. Interestingly the last two *d*-values were assigned to the two different LDH phases namely *d*_3_ - Zn0.67Al0.33–NO_3_ and *d*_2_ - Zn0.75Al0.25–NO_3_. The *d*_3_ value was comparable to the results obtained in (Salak et al., 2012), while the *d*_2_ value corresponds to the phase in which the ratio of Zn/Al cations increased. The difference between the two phases was due to different tilt angles of NO_3_^−^ anions against the layer plane in the LDH gallery with the tilt angle being smaller for the *d*_2_ phase and higher for the *d*_3_ phase. The d_1_ LDH phase having the smallest interlayer distance was ascribed to the carbonate intercalated LDH that forms when the solution contained dissolved CO_2_. The paper states that there were no purification steps performed to remove an excess of CO_2_ thus its intercalation into the LDH gallery as carbonate anions could affect the synthesis. SEM observations revealed a step-by-step evolution of the surface microstructure starting from the appearance of Al hydroxide film on top of the zinc surface, followed by gradually appearance of footprints of zinc oxide interweaved with the crystalline network that could correspond to the *d*_1_ LDH phase (Figure 6B). It was proposed that the *d*1 phase served as nucleation site for the growth of the *d*_2_ and *d*_3_ phases.

The growth mechanism suggested in the work (Mikhailau et al., 2019) emphasized several key processes that involved electrochemical oxidation/reduction reactions of zinc (1), nitrate (2) and oxygen (3) and chemical precipitation at the zinc surface described below.

$$Zn\to {\;Zn}^{2+}+2e^{-}$$ (1)

$${NO}_{3}^{-}+H_{2}O+2e^{-}\to NO_{2}^{-}+2OH^{-}$$ (2)

$$O_{2}+2H_{2}O+4e^{-}\to 4OH^{-}$$ (3)

$${Al}^{3+}+3OH^{-}\to Al{\left(OH\right)}_{3}↓$$ (4)

These processes have been experimentally confirmed by analytical measurements of concentrations of the respective species (Figure 6C) and by EDS analysis of the surface at the initial immersion time that suggested the presence of Al oxide/hydroxide film. The scheme presented in Figure 6D shows the main steps by which the LDH growth process occurs namely oxidation-reduction processes that give rise to pH increase, deposition of Al hydroxide layer and gradual growth of LDH. The chemical equation of LDH precipitation is presented in Eq. 5 and involves soluble species of zinc and aluminum that form LDH in the narrow pH region on Pourbaix diagrams as proposed in (Mikhailau et al., 2019).

$$2\;Zn{OH}^{+}+{NO}_{3}^{-}+Al{(OH{)}_{4}^{-}+mH_{2}O\to {[Zn}_{2}Al(OH)}_{6}{]}^{+}{NO}_{3}^{-}\times mH_{2}O↓$$ (5)

Anionic exchange properties of such LDH films grown on zinc and in particular changes in crystalline lattice and kinetics of anionic exchange processes have been explored in the following works (Bouali et al., 2020a; Iuzviuk et al., 2020). The XRD analysis was the main method for evaluating the changes in the crystalline structure of the LDHs and was performed at the PETRA III synchrotron radiation source (DESY, Hamburg, Germany) with an X-ray energy of 25 KeV. The diffraction patterns were collected from the samples coated with LDH films in the *in-situ* flow cell that allowed monitoring changes by the minute in the crystalline lattice of LDH during the anion exchange process. Several water-based solutions relevant for corrosion applications containing anionic species such as chloride (Cl^−^), sulphate (SO_4_^2-^) and vanadate (VO_x_^*y*−^) were used for *in situ* intercalation experiments.

**FIGURE 7:**
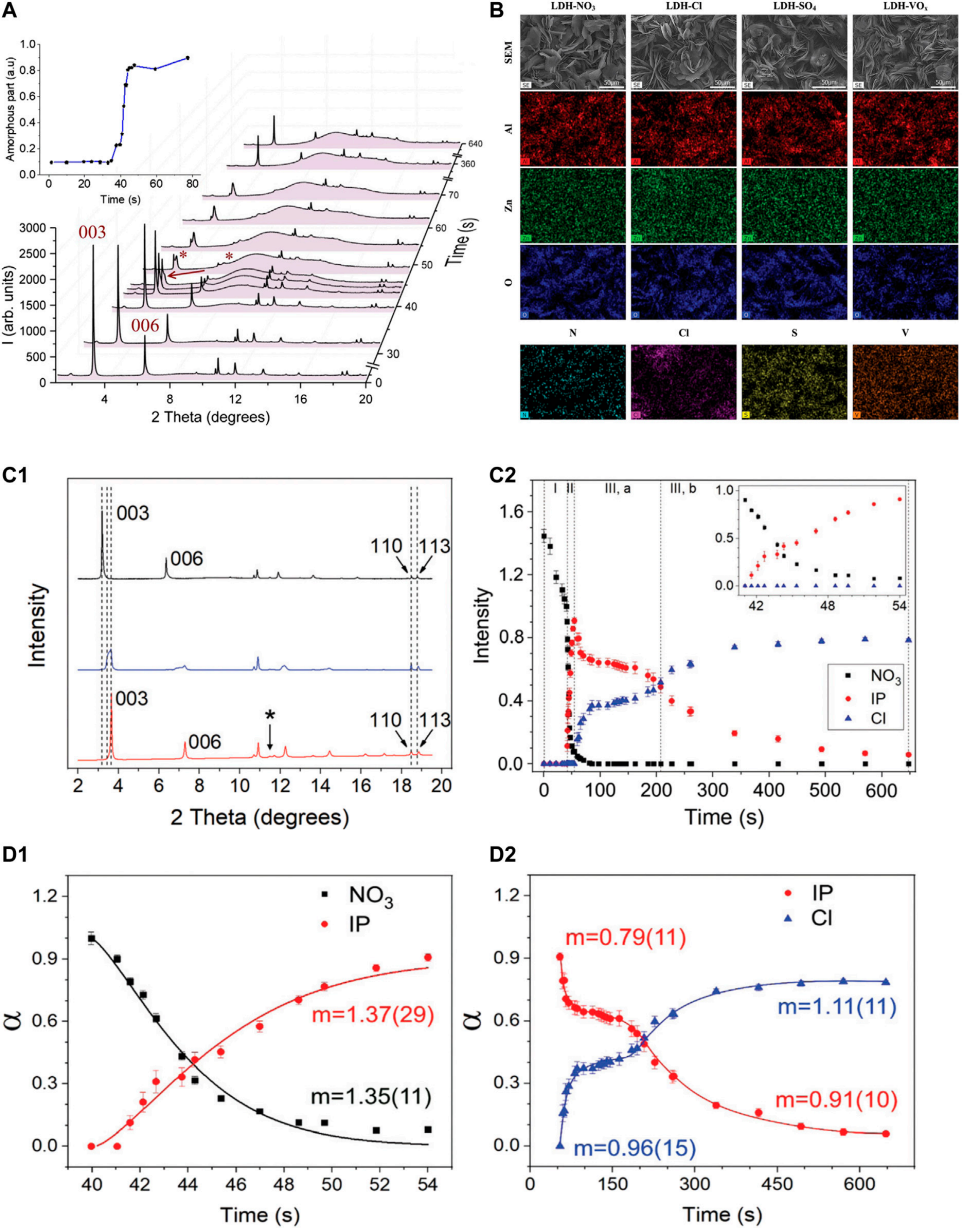
XRD patterns **(A)** for Cl^−^ intercalation process on Zn coated with LDH (Bouali et al., 2020a); SEM-images and EDS maps **(B)** of LDHs with NO_3_^−^, Cl^−^, SO_4_^2-^, VO_x_^*y*−^; **(C1)** XRD patterns of initial LDH-NO_3_ (black), state of coexistence of initial and final phases (blue) and final LDH-Cl (red) phases; **(C2)** time evolution of the integral intensity of 003 peaks of the initial (LDH-NO_3_, squares), intermediate (IP, circles) and final (LDH-Cl, triangles) phases; Kinetic dependences of the degree of substitution of NO_3_^−^ by Cl^−^ in Zn-LDH: **(D1)** interval II: release of NO_3_^−^ (black squares), IP formation (red circles); **(D2)** interval III: IP decrease and formation of the final crystalline phase with Cl-intercalated (blue triangles). Solid lines are the fittings of the AE model. Reproduced from (Iuzviuk et al., 2020) with permission from the Royal Society of Chemistry.

Figure 7A presents XRD patterns taken from the zinc surface coated with LDH before (0 time) and during immersion in Cl^−^ containing solution (Bouali et al., 2020a). The intensities of (003) and (006) Bragg peaks decrease upon exposure to the solution and new Bragg peaks associated with a new LDH phase containing Cl^−^ appear after an induction period. In addition to the changes in the crystalline lattice of LDH, a strong signal of the amorphous phase appears on diffractograms that can be seen on the inset in Figure 7A. Such phase was ascribed to scattering from water, though decomposition of the main LDH phase was also considered as a possible contribution to the amorphous halo. The kinetics of the intercalation process with other anions like SO_4_^2-^ and vanadate VO_x_^*y*−^ have been studied in (Iuzviuk et al., 2020) using the same methodology as presented above. An extensive assessment of LDH microstructure and composition revealed little changes in the morphology of LDH films before and after the anionic exchange process which was confirmed not only by the shift of Bragg peaks (003) and (006) positions but also *via* X-ray maps of different species (Figure 7B). The anionic exchange kinetics was analysed by the changes of intensities from the (003) basal plane as the most intense peak belonging to the LDH phases as presented in Figures 7C1,C2. For the analysis of the time dependencies of the anionic exchange reactions, the equation of Avrami–Erofeev (AE) 6) has been utilized.

$$a\left(t\right)=1-\exp\left[-k{\left(t-t0\right)}^{m}\right]$$ (6)

Where (α) is the reaction extent obtained as the ratio of the integral intensity of (003) reflections at a time (*t*) to the maximal integral intensity, (*t0*) is the time of induction period, (*m)* is a single reaction index that combines the nucleation rate law with the growth mechanism of the nucleus, and the parameter (*k)* characterizes the reaction rate. The fitting of the kinetic dependencies has been done using the AE equation and the results are presented in Figures 7D1,D2. The obtained values of the (*m*) parameter for the disappearing phase with NO_3_^−^ suggested that the reaction mechanism is a two-dimensional diffusion-controlled one including a decelerated nucleation (Figure 7D1). Likewise, the formation of the phase containing Cl^−^ anions are better described by the AE equation than the others, though the values of (*m*) suggest a one-dimensional diffusion-controlled reaction taking place with a decreasing rate of nucleation. Other anions such as SO_4_^2-^, VO_x_^*y*−^ reveal slower kinetics of anionic exchange compared with Cl^−^ ones. Moreover, the intercalation process of SO_4_^2-^ corresponds to a one-dimensional diffusion-controlled reaction with the effect of decelerator nucleation, while the process for VO_x_^*y*−^ is characterized by a two-dimensional diffusion-controlled reaction following instantaneous nucleation. This study provided fundamental characterization of the anionic exchange properties of LDH films synthesized on zinc metal surface which opens further applications of such LDH films in the development of intelligent corrosion protection schemes for zinc-based substrates.

**FIGURE 8:**
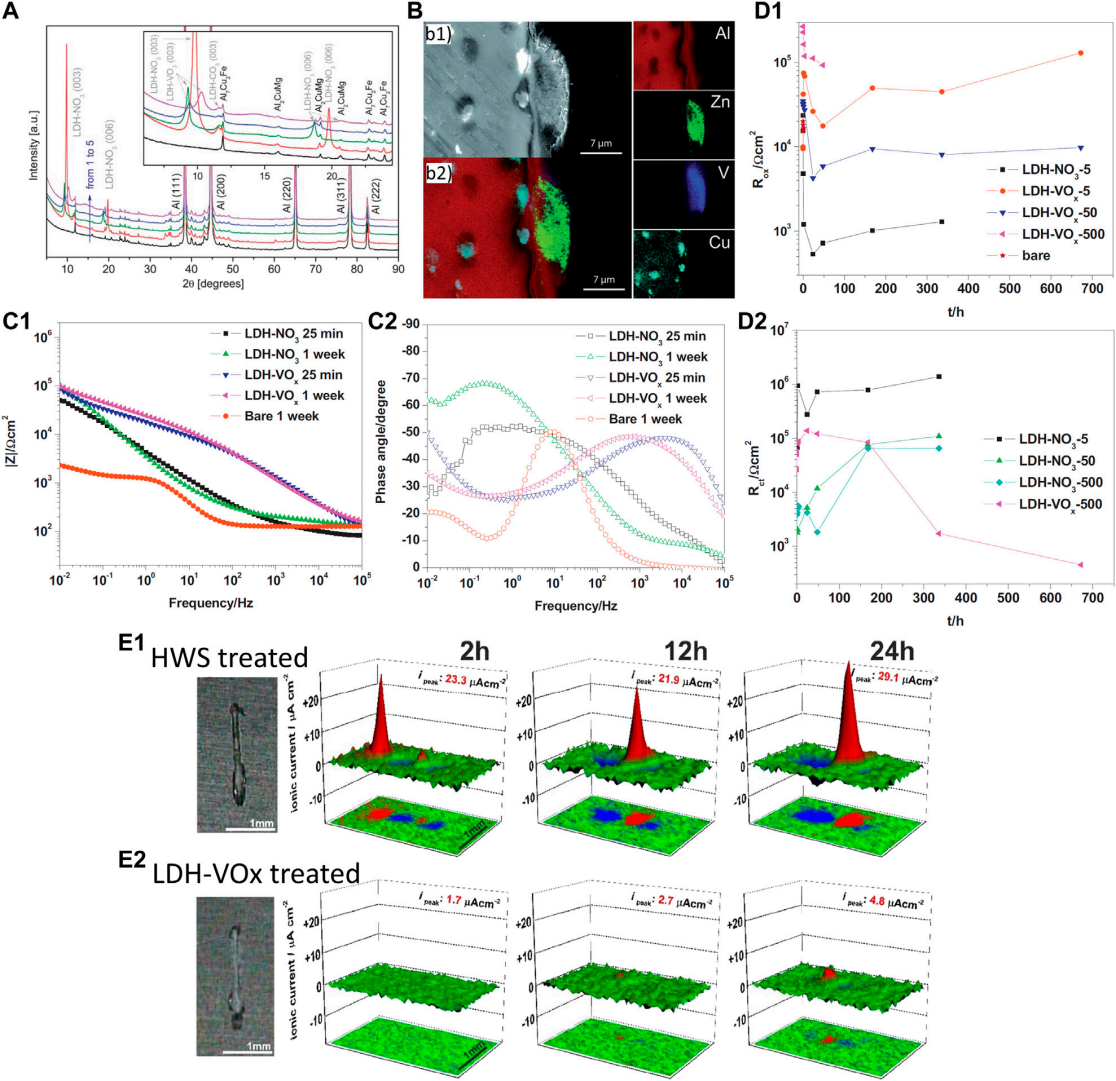
XRD patterns **(A)** of the AA2024 samples: untreated (1), covered with ZnAl LDH-NO_3_ (2), and ZnAl LDH-V_2_O_7_ as prepared (3) or immersed in 0.05 M NaCl for 2 weeks (4) and for 1 month (5). Reproduced from (Tedim et al., 2011) with permission from the Royal Society of Chemistry. The inset shows the patterns at the ranges of (003) and (006) diffraction reflections; SEM images (cross-section) of ZnAl LDH intercalated with vanadates **(B1)** and its corresponding EDS analysis **(B2)**; EIS spectra **(C1,C2)** acquired for AA2024-T3 in 0.05 M NaCl after growth of LDH-NO_3_ and LDH-VO_*x*_ conversion films (prepared with 5 mM Zn^2+^ solution); evolution of *R*_ox_ **(D1)** and *R*_ct_ **(D2)** as a function of immersion time. Reprinted from (Tedim et al., 2014) with permission from Elsevier; Microphotographs, SVET profiles and projection for the samples with HWS **(E1)** and LDH-VO_x_ **(E2)**. Reproduced from (Kuznetsov et al., 2016) with permission from The Royal Society of Chemistry.

As stated earlier in this section various methods for growing LDH coatings have been developed. Nevertheless, the methodologies are sometimes too complex and require harsh conditions, and the LDH films most often contain carbonate, hydroxide, and hydrophobic species that afford passive protection against corrosion. For Mg alloys, the hydrothermal synthesis of LDH is state of the art (Wang et al., 2010; Ye et al., 2018). However, it was shown that the LDH films intercalated with nitrate anions are successfully grown on aluminum alloys at a temperature <100 °C in simple bath chemistry (Tedim et al., 2011; Tedim et al., 2014). Cleaned AA2024-T3 plates were immersed in the solution of Zn(NO_3_)_2_ in the neutral pH range for a few hours at T < 100 °C. Afterwards, the plates were washed with ultrapure water and ethanol and dried in air and the obtained samples were designated as ZnAl LDH–NO_3_. The anionic exchange process for vanadate anions was carried out in 0.1 M NaVO_3_ solution at T < 50°C for a few hours (Tedim et al., 2011). The vanadate species have been chosen as they offer superior corrosion inhibiting performance. Figure 8A displays diffractograms of the AA2024 substrates with and without LDH coatings. The distinction between the ZnAl LDH-NO_3_ (2) and ZnAl LDH-V_2_O_7_ (3) is visible since the reflexes (003) and (006) are shifted to lower 2*θ* angles as was shown in a previous study (Salak et al., 2010). The patterns also reveal shifts in positions and some broadening of (003) reflexes after immersion in 0.05 M NaCl solution for 2 weeks (4) and 1 month (5). To understand how much vanadates anions have been substituted by Cl^−^ ones the changes in basal spacings were analyzed by a superlinear function (Tedim et al., 2011), which suggested that approximately 50% and 90% of the vanadates intercalated in the LDH were substituted by the end of 2 weeks and 1 month of immersion respectively. The study also revealed that the intermetallics such as S-phase (Al_2_CuMg) were the preferred places for the growth of LDH as can be seen on the cross-section SEM picture and EDS maps of different elements (Figure 8B). The authors proposed that at the places of intermetallics aluminum oxide/hydroxide films is broken and dissolution of aluminum at such places is higher, which explained thick LDH deposits found preferentially on S-phase particles (Figure 8B).

The follow-up study (Tedim et al., 2014) explored in more detail the corrosion protective capabilities of the LDH coatings grown on AA2024 substrates. A broad range of concentrations of Zn(NO_3_)_2_, i.e., 5 mM 50 mM and 500 mM was used for LDH growth producing the coatings abbreviated respectively as follows LDH-NO_3_-5, LDH-NO_3_-50 and LDH-NO_3_-500. The anionic exchange process was done similarly as described in the paragraph above, and the resulting samples were named LDH-VO_x_-5, LDH-VO_x_-50 and LDH-VO_x_-500. Initially, the microstructural information was obtained from the coated samples. It appeared that the LDH coatings became denser with increasing the concentration of Zn(NO_3_)_2_. Following that, the assessment of corrosion protection was done using the EIS method during immersion in 50 mM NaCl solution and some results are presented in Figure 8C. Interestingly, although all the coatings presented a higher impedance at a low frequency than the bare uncoated alloy, the coatings containing vanadate (LDH-VO_x_) displayed higher barrier properties than the nitrate-based coatings (LDH-NO_3_) (Figures 8C1,C2). The impedance spectra were fitted to appropriate equivalent circuit models describing in a physical way the phenomena occurring in the coatings, oxide films and at the electrode/electrolyte interface. the protective efficiency of the denser coatings was much inferior to that of the thinner coating. Kinetic dependencies of oxide resistance (*R*_ox_) and charge transfer resistance (*R*_ct_) are presented in Figures 8D1,D2. These parameters represent the corrosion protection efficiency and the higher the values the better the protection of a system. The results revealed that only the thin coatings prepared LDH-VO_x_-5 and LDH-VO_x_-50 show the best performance in terms of active corrosion protection since the *R*_ct_ was too high and thus was not included in Figure 8. Although LDH-NO_3_ showed some protection according to electrochemical results, the optical picture taken from the surface did show significant corrosion. These studies provided a solid ground for the development of intelligent complex protection systems involving a combination of anodic oxide with LDH layers, and sol-gel coatings with LDH layers that will be briefly discussed below.

The aerospace industry utilizes state-of-the-art conversion treatments of the aluminum alloys employing tartaric sulfuric acid anodizing (TSA), phosphoric sulfuric acid anodizing (PSA) processes as an example (Martínez-Viademonte et al., 2020), which exclude highly carcinogenic Cr(VI) species formerly employed in the chromic acid anodizing (CAA) process (Critchlow et al., 2006). The latter process passivates the surface of aluminum alloy and enhances its active corrosion protective properties due to the presence of Cr(VI) species. However, the TSA and PSA processes provide only passive protection and in case of damage to the coating, uncontrolled corrosion will start in the place of the defect. Since anodized coatings possess intrinsic porosity, it opens a pathway to seal the pored with LDH. The development of LDH coating capable of delivering corrosion inhibitors in the defects of the TSA anodic layer was presented in ref. (Kuznetsov et al., 2016). The AA2024 substrate was cleaned using a standard 3-step pre-treatment employed in the aerospace industry and anodized at 14 V for 25 min in a tartaric/sulfuric acid bath containing 0.53 M C_4_H_6_O_6_ and 0.46 M H_2_SO_4_ at 37°C. The anodized samples were immersed for 30 min in a solution of 0.01 M Zn(NO_3_)_2_ and 0.06 M NH_4_NO_3_ with a pH of 6.5 and a temperature of 95°C. Afterwards, the samples were rinsed with deionized water and dried in air and the samples were designated as LDH-NO_3_. An additional anionic exchange process was carried out in the solution of NaVO_3_ at different immersion times such as 30 min and 60 min, which produced LDH coated samples with incorporated vanadate anions (LDH-VO_*x*_). Finally, an additional process such as hot water sealing (HWS) was applied to make an adequate comparison for the LDH-coated samples. The samples containing LDH-VO_*x*_ revealed better corrosion performance in EIS tests and also demonstrated efficient active corrosion protection in local measurements of ionic currents performed by the SVET technique on the scratched surface during immersion in 0.05 M NaCl solution (Figures 8E1,E2). Ionic currents normally increase due to uncontrolled corrosion at the metal surface. However, in the case of LDH-VO_*x*_ coating, the SVET maps showed small and almost unchanging corrosion activity (Figure 8E2) which was attributed to the active role of vanadate species released from the coating.

**FIGURE 9:**
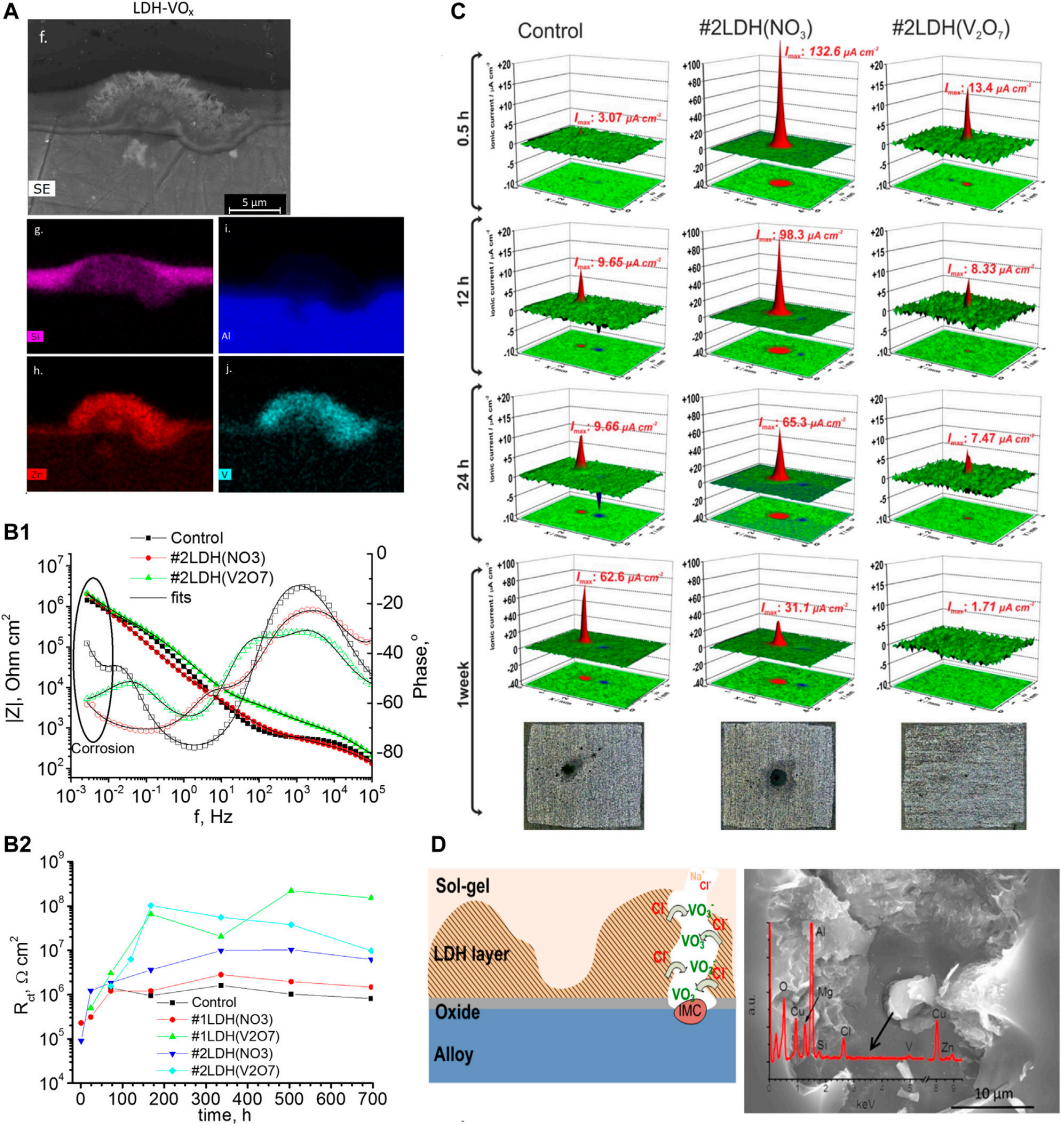
**(A)** SEM micrographs and EDS elemental maps of #1LDH-V_2_O_7_ cross-section; **(B1)** EIS spectra of Control, #1LDH-NO_3_ and #1LDH-V_2_O_7_ coatings after 14 days immersion in 0.5 M NaCl solution; **(B2)** evolution of charge transfer resistance *R*_ct_ parameter during immersion; **(C)** SVET ionic currents maps during immersion in 0.05 M NaCl; **(D)** mechanism of corrosion inhibition. Reproduced from (Yasakau et al., 2018) with permission from Elsevier.

Another work studied the protective capabilities of bi-layer protective systems comprised of LDH conversion treatments and sol-gel pre-treatment in corrosion protection of AA 2024 (Yasakau et al., 2018). The main idea was to explore long-term corrosion protection and active corrosion protection with general (EIS) and local (SVET) electrochemical methods of analysis. Before LDH growth, the alloy samples were cleaned with two processes: a simplified procedure involving 0.1 M NaOH and 0.1 M HNO_3_ and denominated as (#1), and a three-step industrial process denominated as (#2), more details in (Yasakau et al., 2018). The LDHs conversion treatment was done in the same way as reported above (Tedim et al., 2011) and involved initially the preparation of nitrate containing LDH in which nitrate anions were consecutively substituted by vanadates. Subsequently, a model hybrid sol-gel formulation was applied, and three coating systems were produced, namely the sol-gel coatings only (Control), the sol-gel coating atop an LDH layer with intercalated nitrates (#1LDH-NO_3_), and sol-gel coating atop an LDH layer with intercalated nitrates (#1LDH-V_2_O_7_). Figure 9A shows a selected cross-section of the coating #2LDH-V_2_O_7_ and corresponding signals from Si, V and Zn which point out to the place of the sol-gel and LDH respectively. The EIS results revealed rather complicated spectra (Figure 8B1) which were fitted with equivalent circuit models considering the properties of the barrier coating, oxide layer and corrosion process. The latter was characterized by charge transfer resistance (R_ct_) and its kinetics were plotted in Figure 9B2. In comparison to the Control and #1LDH-NO_3_ or #2LDH-NO_3_ coating systems the #1LDH-V_2_O_7_ and #2LDH-V_2_O_7_ ones revealed the highest *R*_ct_ and consequently the best performance during immersion in 0.5 M NaCl. Moreover, it also revealed active corrosion capabilities and self-healing events that were picked up by SVET measurements displayed in Figure 9C. In addition, the results pointed out that the LDH-NO_3_ coating also improved corrosion protection according to the EIS results, which is similar to the results of the previous study (Tedim et al., 2014). Finally, the corrosion protection mechanism involved the release of vanadate anions which acted as the corrosion inhibitor toward the localized corrosion involving the intermetallics and aluminum matrix (Figure 9D).

In a more recent work, authors grew spatially resolved LDH film on AA2024-T3 substrate and modified the resulting film, first by intercalating MBT, and then by modifying the resulting film with an hydrophobic silane (Neves et al., 2019). The intercalation of MBT led to a decrease in the thickness of the LDH film, while the functionalization of LDH-MBT with silane increased the water contact angle almost into the superhydrophobic domain (144°). Moreover, the resulting film was found to exhibit high corrosion resistance and anti-microbial action.

#### 2.2.3 Use of LDH for controlling the corrosion of steel in reinforced concrete

Another area of research where LDH are being tested is in concrete, particularly in the corrosion control of the reinforcing steel. Concrete is the world’s most used construction material. The cement production is responsible for 8% of the anthropogenic CO_2_ emissions (Wu et al., 2022). Hence, extending the lifetime of concrete structures has a societal, economic, and environmental impact. Concrete alone has a very high durability but once steel in placed inside (reinforced concrete) its lifespan is significantly reduced (Bertolini et al., 2013). This is due to the corrosion of steel inside concrete. At the beginning, the steel bars in concrete are in the passive state due to the high pH of concrete (12.5–13.5). With time, atmospheric CO_2_ penetrates the concrete pores and reacts with Ca(OH)_2_ leading to the carbonation of concrete, with the formation of CaCO_3_ and a local decrease in pH. When the carbonation front reaches the reinforcing bars the steel passivation is lost, and corrosion can start. The corrosion products of iron are more voluminous than the metal. The extra volume creates internal stresses leading to cracking and spalling of the concrete cover. This exposes the steel bar to the environment further accelerating the corrosion process. Consequently, there is a great economic and technological interest in controlling this degradation phenomenon.

It is in this context that LDH are being tested to verify their ability to increase the service life of concrete structures. This can be achieved using two properties of LDH referred above, namely, the release of intercalated corrosion inhibitors and the capture of aggressive anions (chloride for example). The first reported attempt to counteract the ingress of chloride ions in concrete using LDH was made in 2003 with a CaAl-NO_2_ added to the concrete mixture (Tatematsu and Sasaki, 2003). The action of the LDH involved the ionic exchange between the nitrite in the interlayer with the chloride inside the concrete. As a result, in the concrete pore solution the amount of free chloride decreased and the concentration of corrosion inhibitor (NO_2_^−^) increased, both contributing to the enhanced corrosion resistance of the steel reinforcement. At about the same time, in 2004, CaAl LDH with intercalated organic acids were used for controlling the cement hydration kinetics (Raki et al., 2004). This is another application of LDH in concrete, together with its use for improving the mechanical properties (compressive strength and flexural strength). Notwithstanding, most of the research is dedicated to the corrosion protection of the steel rebars in concrete with a significant number of papers being published in the last years. An overview of these works can be found in few reviews (Yang et al., 2013; Mir et al., 2020; Yang et al., 2021). There it becomes clear that MgAl is the most studied LDH with cement, mortar, or concrete, followed by CaAl, and then by modified or unmodified hydrotalcite. The main intercalated ions are nitrate, carbonate, and chloride.

**FIGURE 10:**
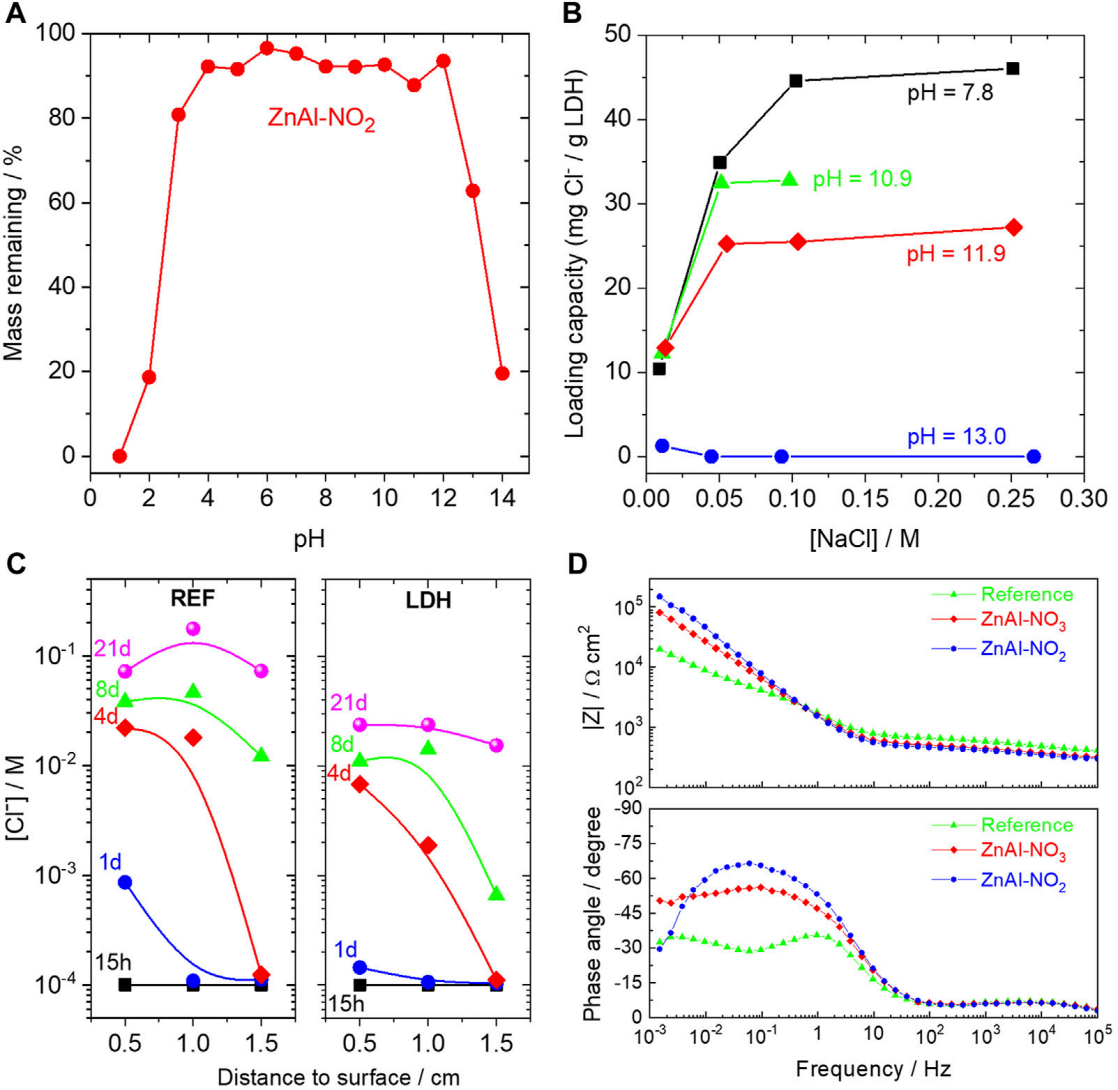
**(A)** LDH powder remaining after 1 month of immersion in water with pH from 1 to 14; **(B)** Chloride loading capacity of ZnAl LDH-NO_2_ at different pH; **(C)** Profiles of chloride ion inside mortars with LDH and without (REF) exposed to 0.5 M NaCl; **(D)** Impedance response of mortars with steel bar immersed in 3.5% NaCl without and with 0.3% (2% with respect to cement) ZnAl LDH-NO_3_ or ZnAl LDH-NO_2_ LDHs.

Recently, we have studied ZnAl LDH in mortar samples. This LDH has been studied in our group before, as an additive in coatings for corrosion control or as surface pre-treatment. This time the objective was to assess the advantage of using this LDH with intercalated nitrate or nitrite to extend the lifetime of reinforced concrete (Gomes et al., 2020). ZnAl-NO_3_ works as a chloride trap while ZnAl LDH-NO_2_ has the additional ability of releasing a corrosion inhibitor. The work analysed the stability of the LDH in different pH, the ability to capture chloride in solutions of different pH, and the impact in slowing the ingress of chloride and in preventing the corrosion of steel bars inside mortar. Figure 10 summarizes the main findings. One important feature to consider is the stability of the LDH in the service environment. Figure 10A shows that ZnAl LDH is unstable at low and high pH. It dissolves in the high pH values found in concrete (e.g., about 40% in pH = 13). XRD analysis of the remaining powder (Mir et al., 2021) showed the appearance of peaks of aluminium and zinc oxides and hydroxides above pH = 12.5, and the absence of LDH structure above pH 13.5. This has a great impact on the expected action of LDH inside concrete because the pH of concrete lies precisely within these values. Equally important is the capability of the LDH to capture chloride and the effect of pH in the process. This is shown in Figure 10B where a substantial amount of chloride is captured by ZnAl LDH in near neutral solutions. However, as the pH increases the capturing capacity declines. At pH 11.9 the capacity is reduced to about 55% and is lost at pH = 13. The reason is the increasing concentration of hydroxide ion which competes with chloride and is preferentially captured. Again, this occurs at the high pH characteristic of concrete. The low LDH stability and its weak capacity to capture chloride at high pH indicate that this might be a bad choice for use in concrete. However, the results of embedded chloride sensors inside mortar with and without LDH revealed a smaller amount of chloride ions in the pore solution of the mortar with LDH, as shown in Figure 10C. This clearly indicates a positive effect of the addition of the ZnAl LDH. Moreover, corrosion tests made on steel bars embedded in mortars containing either no LDH (reference samples) or ZnAl-NO_3_ or ZnAl-NO_2_ LDH, showed better corrosion resistance on samples with LDH, higher for the one containing corrosion inhibitor. The impedance spectra presented in Figure 10D were measured after 70 days of immersion in 3.5% NaCl. The reference sample showed a lower charge transfer resistance and presence of a diffusional process. In contrast, much higher impedance was measured on the other two samples, with a capacitive response dominating at the middle and lower frequency ranges, indicative of passivation of the steel bar. This can be attributed to the chloride capture by ZnAl-NO_3_ and the capture + inhibition by ZnAl-NO_2_ (Gomes et al., 2020). However, these results seem to be inconsistent with the previous observations of partial dissolution and low Cl^−^ capture capacity of the ZnAl LDH at high pH. A tentative explanation is that the LDH dissolution brings local increment of aluminium ions which in the presence of high chloride concentration might precipitate as Friedel’s salt. This could explain the sequestration of chloride ions. The inhibitor is released when the LDH structure is destroyed and becomes readily available to act. Work is ongoing to verify this hypothesis.

The above findings put in perspective many of the works published so far since most of them were done in simulated pore solution (typically saturated calcium hydroxide but in some cases near neutral solutions), just a few in mortar and only one in real concrete (Tatematsu and Sasaki, 2003). It becomes clear that stability and ionic exchange tests must be performed in conditions close to the service environment. Ultimately, only tests in real mortar or concrete samples will allow the correct assessment of the practical use of these new materials.

#### 2.2.4 LDH in corrosion detection

In the field of corrosion protection, there is a need to extend the service life of structures by not only improving the anti-corrosion protection but also provide additional functionalities which may somehow aid on the earlier detection of corrosion onset. While most works published in the literature have focused on the use of pH indicators and fluorescent molecules (Li and Calle, 2008), there is also the possibility to use complexing agents that can react with corrosion products and thereby signal corrosion processes.

In a recent work by Wilhelm and co-workers (Wilhelm et al., 2020), hexacyanoferrate salts were investigated as indicators for early detection of corrosion in carbon steel. Both syntheses of MgAl and ZnAl LDH intercalated with hexacyanoferrate salts were attempted, but only MgAl LDH was found suitable for intercalation of this anion, as Zn in ZnAl LDH tends to react with hexacyanoferrate and form a brownish insoluble precipitate. XRD and FTIR revealed the presence of different forms of hexacyanoferrate within LDH, which can both react with Fe cations and form intense blue precipitates (Turnbull’s Blue and Prussian Blue). Release studies performed under different conditions showed that hexacyanoferrate is preferentially released under high NaCl concentrations. In addition, electrochemical studies (potentiodynamic polarization and EIS) demonstrated that corrosion of carbon steel increases when this anion is fully available in solution, with this effect being minimized when the anion is intercalated within LDH. Visual analysis of exposed carbon steel substrates to LDH in aqueous NaCl solution and simulated FeCl_2_ solution revealed the formation of the blue precipitates, which underlines the success of this approach. The next step was to carry out similar studies in coatings modified with LDH-hexacyanoferrate. Indeed, the combination between electrochemical and visual analysis of the substrate shows that the appearance of dark blue spots at the metal coating interface is associated with the decrease of overall impedance and initiation of corrosion processes, before the appearance of iron-based corrosion products (Sushkova et al., 2021).

#### 2.2.5 Environmental and biological aspects associated with LDH

One of the requirements associated to the use of micro and nanocontainers is that these systems can not pose environmentally negative effects as those associated with Cr(VI)-derived species. They shall also contribute for a lower availability of the active species in the surrounding media when they are not necessary. This can contribute for a long-term protective action, while at the same time reducing the harmful effects associated with intercalated toxic species.

Having carried out several works on the assessment of LDH-MBT and knowing that MBT displays biocidal properties, we decided to investigate the antibacterial effect of MBT when intercalated in Zn-Al LDH (Kuznetsova et al., 2017). Different conditions of salinity (1, 2 and 3% NaCl) and pH (4, 5, 6 and 7) were used and the biological effects of MBT immobilized and released from LDH were monitored using an assay based on bacterial bioluminescence of either *Allivibrio fischeri* or a recombinant strain of *Escherichia coli*. While release studies carried out by UV-Visible spectrophotometry run under similar conditions of salinity and pH indicated that MBT is released swiftly, with high extent of release in 3% NaCl and under alkaline conditions, the biological studies revealed a more pronounced toxic effect (decrease of cell viability) in 1% NaCl solution. The differences observed between extent of released MBT and toxicity measured imply that the toxic effect induced in the bacteria was a function of both extent of released MBT and optimal salinity conditions for the growth of model bacteria. Also relevant is the fact that ZnAl LDH-NO_3_ did not induce any light emission by the bioluminescent bacteria.

The toxicity induced by ZnAl LDH-MBT to the clam *Ruditapes philippinarum* was also studied in a joint work with Martins and Loureiro (Martins et al., 2017b). The results obtained demonstrated that the immobilization of MBT in the LDH provides some protection effect for the clams, reducing both physiological and lethal effects, though still presenting some hazardous effects. The chemical toxicity was ranked as being the highest for MBT and the lowest for LDH. In another work in collaboration with Martins and Loureiro, zinc and copper pyrithione booster biocides were immobilized into Zn-Al LDH and the anti-fouling efficacy and toxicity of the materials obtained was tested using green microalgae *Tetraselmis chuii* (non-target species) and the diatom *Phaeodactylum tricornutum* and the mussel *Mytilus edulis* (target species) (Avelelas et al., 2017b). When comparing the free form of the biocides, biocide-free LDH and biocide-containing LDH, the results obtained indicated that incorporation of zinc pyrithione in LDH was found to be an environmentally friendly way of using the biocides, without compromising the antifouling efficacy compared to the free biocide. In another joint work with Martins and Loureiro, and with Benayahu and co-workers (Gutner-Hoch et al., 2018), the anti-macrofouling efficacy of Zn-Al LDH loaded with zinc and copper pyrithione was investigated. In this study, adult mussels from Atlantic Ocean, Mediterranean Sea and Red Sea, together with larvae of bryozoan *Bugula neritina* from Mediterranean Sea and Red Sea were used. The results obtained demonstrated that LDH with zinc pyrithione could be used in lower quantities with respect to other compounds used currently to render anti-macrofouling effects and the fact that LDH can provide controlled release of the compound, thereby allowing targeted foulers to be tackled while still limiting the release of the active compounds to ambient waters. Similar trends were found in another study conducted on larval stage of the brine shrimp *Artemia salina*, and on embryonic European purple sea urchin *Paracentrotus lividus* (Gutner-Hoch et al., 2019). Overall, the results support the use of LDH as eco-friendly nanomaterials for development of high-performing protective coatings.

## 3 Exploring LDH *via* computational tools

### 3.1 The role of computational tools

During the last few years, our research team has been using different computational tools to explore the structure, reactivity, and properties of LDH at different scales. As highlighted above, LDH not only play a key role in the field of corrosion protection as nanocontainers through hosting corrosion inhibitors or molecules capable of signaling corrosion but have been finding application in a wide range of fields. For this reason, some of the conclusions obtained using computational tools to gather insights into the structure and properties of LDH, their interlamellar reactivity and dynamics, have also been important to other fields.

Unfortunately, information about the structure, stability and kinetics of these bulk systems is difficult to obtain by experimental techniques alone, despite some notable efforts (Kameda et al., 2021), due to their high degree of complexity, regarding different parts of system, notably, 1) the order, types of atoms and defects of the cationic layer; 2) the orientation, conformation, reactivity, and solvation degree of the interlayer molecular contents, and 3) the surrounding conditions in terms of types of species, their concentration, pH and other physicochemical properties, which, in turn, can influence the cationic layer and interlayer contents themselves. Therefore, computational, and molecular modelling tools play a key role in the understanding of these systems, providing unprecedented mechanistic details about the different processes and applications in which LDHs are involved, eventually potentiating and accelerating the design of more efficient and sustainable materials.

In this section, we will review recent advances made with the following tools:

1) Hydrogeochemical modeling rendered insights on the chemical speciation of different metal cations in solution during the synthesis of LDH, which leads to its proposal as a promising solution to model and optimize the production of LDH.
2) Density functional theory (DFT) was used to characterize the crystalline structure of LDH, which allowed to understand and simulate their XRD spectra, while also explaining the shape of LDH particles, the exfoliation of LDH particles to form nanosheets, and the formation of conversion films on top of metallic surfaces.
3) Classical molecular dynamics (MD) simulations allowed to reach larger time and length scales of realistic models of LDHs, which was key to understand the reactivity and role of solvation inside the interlayer galleries.

Some of these works developed in CICECO had a seminal character, which subsequently led to their use and citation by other authors. Therefore, their impact within the LDH community will also be discussed herein.

### 3.2 Hydrogeochemical modeling

Hydrogeochemical modelling can render insights into the chemical speciation of different metal cations in solution. Therefore, this simulation tool was proposed to model, understand, and optimize the synthesis of LDH based materials (Galvão et al., 2016b).

The more traditional method of LDH synthesis usually involves the co-precipitation of the metals in solution to form the cationic layer and the appearance of the first crystallites with the known layered structure, followed by hydrothermal treatment during which the initial material grows into fully formed LDH particles, usually in the form of hexagonal plates (Galvão et al., 2016b, 2017). Usually, a labile anion (nitrate, for example) is intercalated during this step, which can then be replaced by a functional molecule, also in the anionic form, during a subsequent anion-exchange process. Due to the wide range of applications of LDH (Galvão et al., 2020b), often, there is the need to establish the large-scale production of these materials. For this reason, the relation between the physicochemical properties capable of influencing the synthesis (pH, time and temperature), and the crystallite and hydrodynamic particle sizes of the produced LDH were investigated, and subsequently correlated with the concentration of the metals in solution during the synthesis (Galvão et al., 2016b).

**FIGURE 11:**
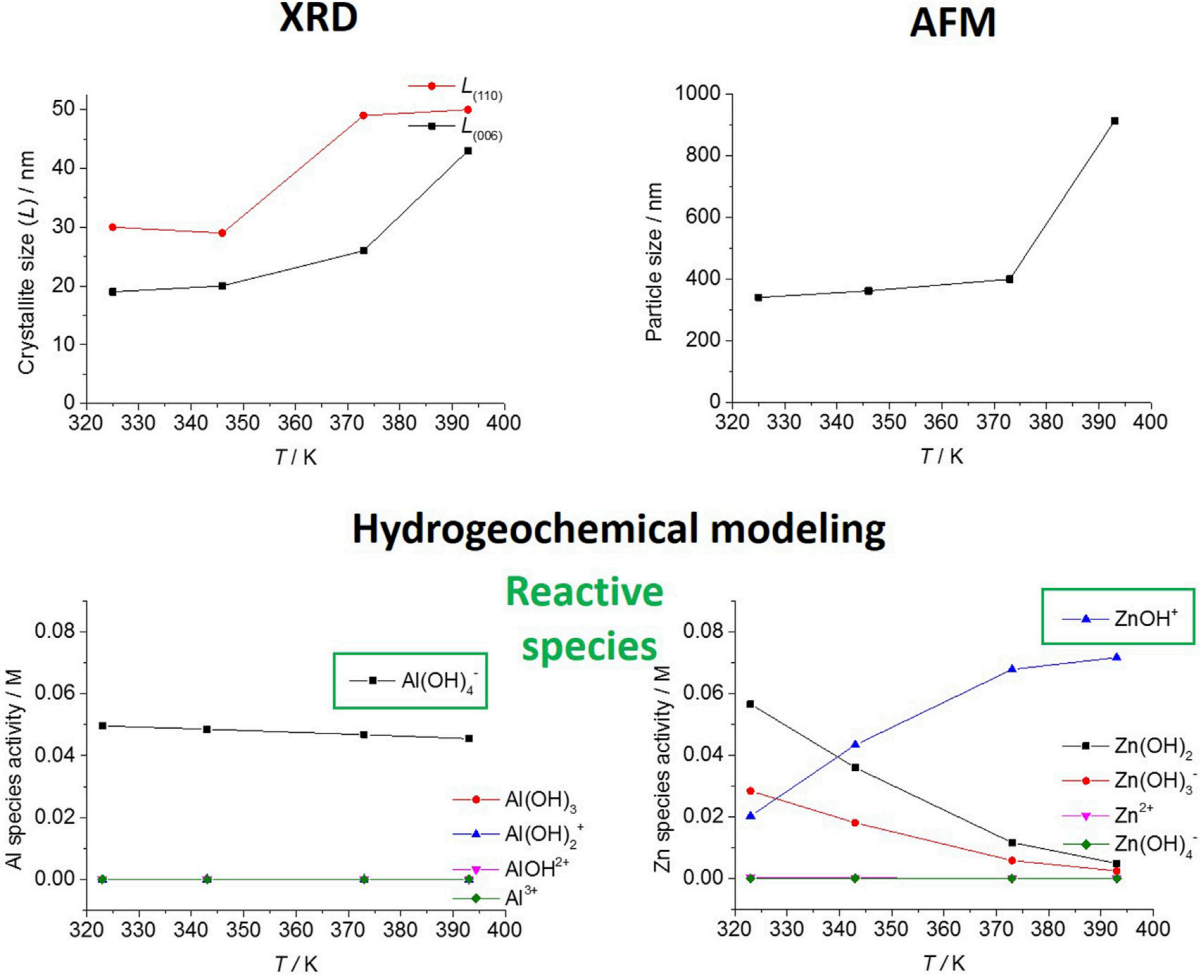
Main reactive species obtained from hydrogeochemical modelling were used to understand the optimal crystallite (XRD) and particle sizes (AFM) for a ZnAl LDH at temperatures in the range 320–400 K and exemplified herein for pH = 10. Images adapted from reference (Galvão et al., 2016b).

An hydrogeochemical modelling software (Parkhurst and Appelo, 2013) was used to estimate the concentrations of different species of aluminum and zinc in aqueous solution under synthesis conditions, considering different temperatures (*T* = 323, 343, 373 and 393 K) and pHs (8.5 and 10), thus allowing to understand the influence of hydrothermal treatment. It was proved that hydrothermal treatments performed at the higher temperatures lead to larger crystallites than treatments at the lower temperatures. Moreover, it was found that the temperature influenced the rate of crystallite reorganization in solution and the equilibrium of aluminum and zinc chemical species. According to the Al and Zn chemical speciation obtained from hydrogeochemical modelling calculations, Al(OH)_4_^‒^ was the only Al species present in solution. In the case of Zn, at pH = 10, Zn(OH)_2_, Zn(OH)_3_^‒^ and ZnOH^+^ were the favored species in solution. However, among these species, only ZnOH^+^ increased its concentration in solution as the temperature increased, thus accompanying the occurrence of larger crystallites and particle sizes. The results shown in Figure 11 supported the important role of Al(OH)_4_^‒^ and ZnOH^+^ in the formation of LDH (Galvão et al., 2016b). Despite the chemical speciation in solution was already proposed by other authors (Zhi and Guo, 2005) as preponderant for the LDH formation mechanism, to the best of our knowledge, for the first time a hydrogeochemical modelling tool was used to help identify the reactive species in solution. The potential of this strategy was recognized by other authors (Conterosito et al., 2018). Subsequently, the identified species were used to propose a reaction mechanism for the LDH formation (Mohedano et al., 2017; Serdechnova et al., 2017). This indicates that hydrogeochemical modelling can be a solution to accelerate the scale-up and increase the efficiency of LDH synthesis.

### 3.3 Density functional theory with periodic models

The crystal structure of LDH can be difficult to determine by XRD due to the poor crystallinity of these materials (Thomas and Kamath, 2006). This makes density functional theory (DFT) calculations with expanded periodic models (a.k.a. periodic model DFT) an ideal technique to gain insights into the inner structural features of new LDH materials. The first DFT studies used LDH structural models based on anhydrous supercells (Trave et al., 2002) or small cluster models (Yan et al., 2008) to reduce the computational cost of the simulations. Afterwards, Costa, Leitão and co-workers designed more realistic periodic models with an ideal size for DFT studies (Costa et al., 2008; Costa et al., 2010; Costa et al., 2012). Inspired on Costa et al. works, a DFT periodic model of the LDH structure was used by us (Galvão et al., 2016b) to unveil the orientation of the nitrate anion inside the interlayer structure of a ZnAl LDH, which was still a matter of uncertainty at the time, regarding the parallel (Xu and Zeng, 2001; Xu et al., 2009) vs. tilted (Costa et al., 2012; Salak et al., 2012) orientation of nitrate anions relative to the cationic layer.

Afterwards, it was explored the structural and energetic driving forces behind the morphology of zinc-aluminum LDH particles, single layer nanosheets and protective conversion films grown on top of aluminum alloys (Galvão et al., 2017).

Periodic model DFT was employed to obtain surface and interaction energies of different orientations of the LDH structure. These results were combined with Atomic Force Microscopy (AFM) and XRD measurements to understand how LDH polycrystalline particles are formed. Fully formed LDH particles, have a plate-like shape, where the lateral size is larger than the particle height. LDH polycrystalline particles seem to be defined by 1–3 crystallites forming the plate height and between 8 and 11 crystallites forming the plate length. Computed surface energies show that particles grow larger in terms of length than in terms of height to minimize the surface energy of particles, since the lateral side of the particles is less stable than the top and bottom of the plates. Moreover, interaction energies show that crystallites have more favorable interaction energies when they interact side-by-side, thus extending the formation of the cationic layers to form the plate shape. On the contrary, crystallites interact less favorably when stacking cationic layers and interlayers one over the other, thus resulting in lower heights than widths. This view of the relation between the plate-like morphology and the inner structure of LDH has already allowed a better understanding of the morphology of LDH based structures in other works (Li et al., 2018; Prestopino et al., 2019; Wu et al., 2019; Aleshin et al., 2020; Roberts et al., 2020).

The literature is vast in what concerns the delamination of LDH when they are dispersed in formamide (Liu et al., 2006; Ma et al., 2006; Han et al., 2008; Abellán et al., 2010; Song and Hu, 2014), even without thermal treatment (Ma et al., 2006). Ma et al. (Ma et al., 2006) proposed a two-stage delamination mechanism for LDH in formamide, i.e., rapid swelling and subsequent slow exfoliation, but confirmation with atomistic detail could be obtained only after computational studies. DFT calculations revealed that formamide conserve or even increase the network of hydrogen bonds in the interlayer, while increasing the distance between cationic layers, when they enter the interlayer to substitute crystalline water molecules, usually intercalated during the synthesis. As the formamide treatment progresses and more formamide molecules substitute water molecules in the interlayer, this is accompanied by a decrease of the layer separation energy for formamide in comparison with water. These structural and energetic tendencies show why formamide can be a good agent to delaminate LDH. The conclusions obtained in that work (Galvão et al., 2017) contributed to subsequent DFT studies on the exfoliation of LDH (Tavares et al., 2020b; Tavares et al., 2021).

**FIGURE 12:**
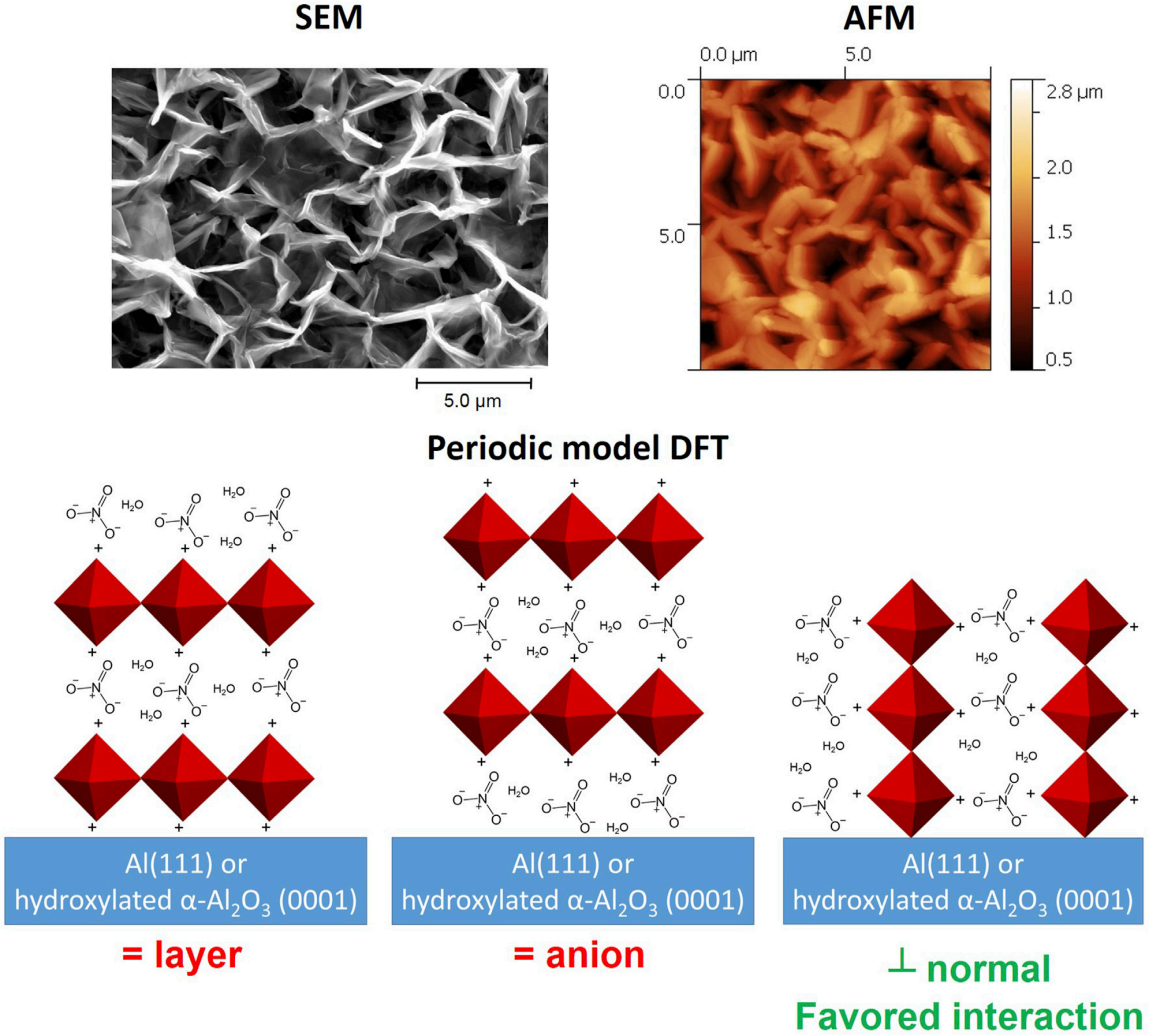
Scheme illustrating the results obtained from periodic model DFT calculations for the interaction between LDH clusters and different aluminum surfaces unveiling the morphological results obtained by SEM and AFM. Images adapted from reference (Galvão et al., 2017).

Regarding LDH conversion films grown on top of aluminum alloys, it is well documented the preference for LDH plates of conversion films to grow perpendicularly to the aluminum substrate, rather than lying flat on top of the aluminum surface and growing in terms of height (Tedim et al., 2011; Tedim et al., 2013; Tedim et al., 2014). For LDH particles and nanosheets it was shown that cationic layers grow longitudinally in parallel to the plate like morphology of LDH. Therefore, in the case of conversion films, the cationic layers should also be perpendicular to the aluminum substrate. However, in order to explore this hypothesis and unveil the relation between atomic structure and morphology of conversion films, the interface between ZnAl LDH-NO_3_ conversion films and the aluminum surface was studied considering the bare metal (Al (111)), more common under extreme pH conditions (Tait, 2012), and the hydroxylated aluminum oxide [α-Al_2_O_3_(0001)] predominant under near neutral pH (Tait, 2012). The conversion films were approximated computationally using LDH clusters with different orientations and sizes, and, for both aluminum surfaces, it was found that the most favorable interaction was with the cationic layers perpendicular to the aluminum surfaces, thus allowing to understand the SEM and AFM results obtained for these systems. In the case of Al (111), the hydroxyl groups of the cationic layer coordinate with the aluminum atoms of the surface, whereas for α-Al_2_O_3_(0001) the metallic atoms of the LDH cationic layer coordinate with the hydroxyl groups resulting from the hydroxylation of the oxide surface in water (Rohmann et al., 2014). These results also elucidate the crystallization mechanism of LDH films. The mechanism has been rationalized by other authors (Guo et al., 2009; Lee et al., 2015) in terms of an initial random grow of crystal seeds. The DFT results point to a preferential orientation of the seeds with the cationic layers normal to the surface even during early stages of crystal grow. After this early stage, even if seeds crystallize away from the surface in other directions, these eventually meet crystals growing with the cationic layers normal to the surface, thus preventing the former from continuing the growing process (Guo et al., 2009; Lee et al., 2015). Part of the experimental and computational results obtained in that work for conversion films (Galvão et al., 2017) are illustrated in Figure 12.

### 3.4 Molecular dynamics

Classical molecular dynamics (MD) has been the main computational approach for the atomistic simulation of LDHs and other clay-like materials (Cygan et al., 2004; Greenwell et al., 2006; Cygan et al., 2009), since it allows to examine models with thousands of atoms in a computational efficient manner. However, despite several works could be found in the literature about the structure and dynamics of the LDH’s interlayer contents (Kim et al., 2005; Lombardo et al., 2005; Kim et al., 2007; Pisson et al., 2008; Tran et al., 2008), it was lacking a straightforward MD procedure, with supercell models, MD steps and parametrization, which could be readily adapted for the simulation of different LDH systems. Therefore, Pérez-Sánchez et al. (Pérez-Sánchez et al., 2018) developed a straightforward framework based on the GROMACS open-source code to perform MD simulations of LDH, which can be easily modified to consider a wide range of inorganic and organic anions intercalated. The developed procedure enables to run simulations for long timescales (>100 ns) with all atomic positions allowed to move freely, while maintaining intact the integrity of the LDH structure. It was demonstrated for different cationic mixtures (e.g., MgAl and ZnAl LDHs) and different intercalated anions (chloride, nitrate and carbonate) that the model and partial charges parametrization allow to study interlayer equilibrium processes in detail. This is an especially important feature of the developed MD framework, since, as far as we know, none of the previous computational models (Rad et al., 2016; Tsukanov and Psakhie, 2016) were able to reproduce realistic experimental conditions for LDH immersed in solution as nanocontainers of active functional species.

**FIGURE 13:**
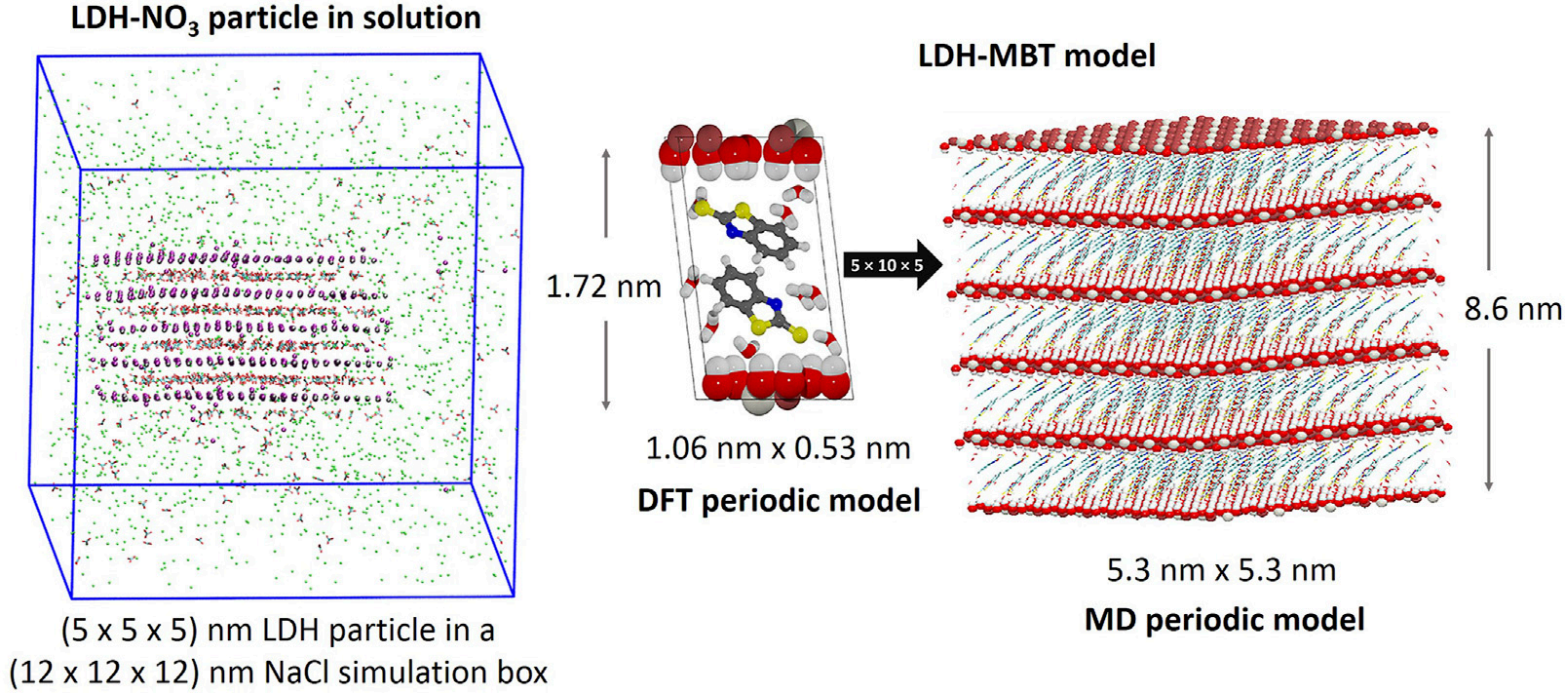
MD simulation snapshot of an LDH-NO_3_ particle in a sodium chloride solution simulating a corrosion medium, showing the stability of the system after 20 ns (left). DFT periodic model, and expanded MD periodic model used for the MD simulation of LDH-MBT (Colour code for spheres: silver, Al; scarlet, Zn; red, O; white, H; grey, C; blue, N; and yellow, S). Images adapted from references (Pérez-Sánchez et al., 2018; Novell-Leruth et al., 2020).

The model considers the periodic expansion of the LDH structure in the directions parallel and perpendicular to the cationic layers (Thyveetil et al., 2008; Lv et al., 2012; Makaremi et al., 2015). Based on the excellent agreement between calculated and experimental data, the model can be considered to simulate the application of LDH in a wide range of fields, having the potential to predict the controlled release of functional anions, such as corrosion inhibitors (Poznyak et al., 2009) or pharmaceuticals (Senapati et al., 2016), the trapping of aggressive or hazardous anions (Lu et al., 2016) or to simulate the influence of pH or other electrolytes on the controlled release of intercalated active compounds (Carneiro et al., 2015). As a test case, LDH particles with intercalated nitrate anions were introduced in a sodium chloride water solution, to simulate an aggressive corrosion environment. Encouragingly, it was found that the layered structure of the LDH was kept stable within the timeframe of a 20 ns MD simulation, as shown in Figure 13, paving the way to study an ion or solvent exchange between the LDH and the surrounding solution that require much longer simulation times.

The model presented for LDH materials is ready to be used with different intercalated anions with the well-known open-source code for MD simulations, GROMACS (Abraham et al., 2015). Therefore, all the necessary parameters and inputs to carry out the MD simulations are available for download (http://sweet.ua.pt/jrgomes/SELMA/MD-LDH/). This framework has already been used by other researchers to model LDH, particularly dealing with the removal of hazardous materials (Zhu et al., 2022), the examination of the undulation of the cationic layers of exfoliated LDHs (Tavares et al., 2020a), following the MD procedure to evaluate the structure of LDH as drug nanocarriers (Pšenička et al., 2020), and taking the partial charge parametrization of the force field to evaluate the hydration states of the interlayer contents (Li et al., 2022).

One key aspect for the application of LDH as nanostructured coating additives for corrosion protection is the choice of the inhibitor to be intercalated. To facilitate this selection, an open cloud database to search for corrosion inhibitors, their respective inhibition efficiencies and measurement conditions was also built (Galvão et al., 2022), and a machine learning approach to evaluate new inhibitors is being developed (Galvão et al., 2020a). One particularly efficient inhibitor is 2-mercaptobenzothiazole (MBT), which was previously studied regarding its tautomeric, acid-base and ion-pair formation equilibrium in aqueous solution (Galvão et al., 2016a), as well as its ability to adsorb onto aluminum surfaces (Galvão et al., 2018). To understand its acid-base equilibrium, conformation and degree of solvation inside the interlayer galleries, the LDH-MBT system was studied by classical MD (Novell-Leruth et al., 2020) using the model presented in Figure 13. The MD model is a 5 × 10 × 5 expansion of the smaller DFT periodic model, thus allowing to simulate more realistic structural features and follow complex dynamic processes. That work showed how MD simulations can complement the experimental characterization of LDHs, providing atomistic and dynamic insights into the interlayer distance and other structural information obtained by XRD and the solvating degree indicated by TGA.

## 4 Summary and conclusion

Since 2004 the SECOP group at University of Aveiro has been heavily involved in searching for new chemical alternatives to the replacement of traditional applications of Cr(VI) in corrosion protection. This went through doped sol-gel coatings, self-assembled networks as nanostructured reservoirs for self-healing anticorrosion pre-treatments, silanes and rare-earth salts as corrosion inhibitors, zeolites and oxide nanoparticles nano/micro reservoirs for storage of corrosion inhibitors, layer by layer assembled nanocontainers, LDHs nanocontainers filled with corrosion inhibitors, etc.

Among these strategies LDH seem very tenable, versatile, and advantageous for corrosion protection applications. Moreover, they are environmentally friendly, accessible, and applicable to a wide range of metal substrates. In our Laboratory we have investigated and developed the synthesis, characterization, and anti-corrosion properties of these materials as nanoparticles (to be used in paints) or as conversion coatings formed directly on metals. The studies mainly concerned the protection of aluminum alloys, in particular AA2024 used in aeronautics. Nevertheless, other substrates were used, as magnesium alloys and galvanized steel and in a few cases steel.

These materials demonstrate very good ion-exchange capacity, which is very useful for corrosion protection applications since the LDH can provide a simultaneous double-function on removing (trapping) aggressive species from the medium and releasing inhibiting species on-demand for protecting the metallic substrate. Moreover, this mechanism often provides a fast protective response (self-healing) conferring active corrosion protection, triggered by presence of aggressive ions (Cl^−^) or mechanical damage, acting in specific sites and avoiding the uncontrolled leaching of the inhibitor, with improvement in the corrosion protection efficiency and increase of the duration of the coatings (Leal et al., 2022).

In the context of corrosion protection, LDH are still in the process of development and some problems must be overcome before taking full advantage of them. In the case of nanoparticles, challenges as the dispersion and the compatibility of the nanoparticles with the coating formulation, amount of nanoparticles introduced, carbonate contamination during LDH synthesis and storing, difficulties to intercalate large anions or neutral molecules, and instability in acidic or very alkaline media, good corrosion inhibitors in certain media, high cost, among others, must be considered.

However, there are a few companies that have dedicated their R&D to focus in this area of expertise. For instance, KISUMA Chemicals (Innovation - Kisuma Chemicals), a world leader in the production of hydrotalcites, possesses a branch specifically devoted to the development and optimization of LDH pigments for corrosion protection. SMALLMATEK Lda. (Smallmatek–Small Materials and Technologies), which was created as a start-up at the University of Aveiro, produces and performs research on LDH additives mainly for the purpose of corrosion protection. Their pigments can be incorporated into coatings and integrated in a multi-level corrosion protective scheme.

The possibility to use LDH nanocontainers as a post-sealing treatment for anodized and plasma electrolytic oxidation (PEO) coatings has generated a lot of interest in the corrosion field. However, the growth on certain PEO coatings remains challenging (Bouali et al., 2020a).

## 5 Future perspectives and outlook

Despite the scientific achievements and promising potential for use of LDH into real coating systems, the work is yet not complete. LDH need to fit into other requirements such as processing parameters, compatibility with other coatings and adhesion performance. These factors can be overcome by further optimization of the LDH both in form of pigments and conversion coatings. It is important to perform more systematic studies on environmentally friendly inhibitors for specific media/metals that could be intercalated in the galleries, since active corrosion protection comes from them.

Overall, any major development in Materials Science in general and Corrosion Science and Engineering in particular, requires the pursuing of new ways of designing materials and testing them: from application of bio-inspired approaches to design new additives and multifunctional coatings, to the use of AI for design and optimization of materials in ways otherwise not accessible to more traditional trial-and-error, incremental approaches. SECOP group is currently carrying out activities in this direction in collaboration with industry, namely by developing organic coatings from bio-based sources combined with LDH-based nanoadditives (COAT4LIFE) with active corrosion protection and corrosion detection functionalities, developing biocompatible surface treatments for Mg alloys used in biomedical implants and by implementing data-driven approaches to find efficient corrosion inhibitors for different metals (DATACOR) to be subsequently loaded into LDH.
